# Multimodal Investigations of Reward Circuitry and Anhedonia in Adolescent Depression

**DOI:** 10.3389/fpsyt.2021.678709

**Published:** 2021-07-23

**Authors:** Benjamin A. Ely, Tram N. B. Nguyen, Russell H. Tobe, Audrey M. Walker, Vilma Gabbay

**Affiliations:** ^1^Department of Psychiatry and Behavioral Sciences, Albert Einstein College of Medicine, Bronx, NY, United States; ^2^Department of Clinical Research, Nathan S. Kline Institute for Psychiatric Research, Orangeburg, NY, United States

**Keywords:** mood disorders, pediatric, fMRI, MRS, RDoC, cytokine, immune, inflammation

## Abstract

Depression is a highly prevalent condition with devastating personal and public health consequences that often first manifests during adolescence. Though extensively studied, the pathogenesis of depression remains poorly understood, and efforts to stratify risks and identify optimal interventions have proceeded slowly. A major impediment has been the reliance on an all-or-nothing categorical diagnostic scheme based solely on whether a patient endorses an arbitrary number of common symptoms for a sufficiently long period. This approach masks the well-documented heterogeneity of depression, a disorder that is highly variable in presentation, severity, and course between individuals and is frequently comorbid with other psychiatric conditions. In this targeted review, we outline the limitations of traditional diagnosis-based research and instead advocate an alternative approach centered around symptoms as unique dimensions of clinical dysfunction that span across disorders and more closely reflect underlying neurobiological abnormalities. In particular, we highlight anhedonia—the reduced ability to anticipate and experience pleasure—as a specific, quantifiable index of reward dysfunction and an ideal candidate for dimensional investigation. Anhedonia is a core symptom of depression but also a salient feature of numerous other conditions, and its severity varies widely within clinical and even healthy populations. Similarly, reward dysfunction is a hallmark of depression but is evident across many psychiatric conditions. Reward function is especially relevant in adolescence, a period characterized by exaggerated reward-seeking behaviors and rapid maturation of neural reward circuitry. We detail extensive work by our research group and others to investigate the neural and systemic factors contributing to reward dysfunction in youth, including our cumulative findings using multiple neuroimaging and immunological measures to study depressed adolescents but also trans-diagnostic cohorts with diverse psychiatric symptoms. We describe convergent evidence that reward dysfunction: (a) predicts worse clinical outcomes, (b) is associated with functional and chemical abnormalities within and beyond the neural reward circuitry, (c) is linked to elevated peripheral levels of inflammatory biomarkers, and (d) manifests early in the course of illness. Emphasis is placed on high-resolution neuroimaging techniques, comprehensive immunological assays, and data-driven analyses to fully capture and characterize the complex, interconnected nature of these systems and their contributions to adolescent reward dysfunction.

## Introduction

Depression in children and adolescents is associated with significant distress, family burden, and functional impairment including academic failure, social dysfunction, and substance use (1). Critically, pediatric depression also significantly increases the risk of suicide, which ranks as the second leading cause of death between ages 10–34 years (2). Recent epidemiological surveys indicate that up to 20% of adolescents experience at least one depressive episode before entering adulthood (3). The prevalence of major depressive disorder also rises sharply during adolescence, with 12-months positivity rates increasing from 2.7% at ages 8–15 years (4) to 8.2% by ages 12–17 years (5). Yet, only one in three depressed adolescents currently receives disorder-specific treatment (6). Adolescent depression is also a strong predictor of depression in adulthood (7–9), which is ranked by the World Health Organization as the leading cause of years lived with disability (10).

Efforts to address this major public health challenge are complicated by the considerable variation in adolescent depression presentation, severity, and especially course. While approximately 40% of depressed youth will fully recover by adulthood (7), others will experience persistent and progressive illness, often despite apparently successful treatment (11–16). For example, Birmaher et al. followed 107 adolescents treated with psychotherapy and demonstrated that, despite high short-term remittance rates of around 80%, 38% of these patients later experienced symptom recurrence (13). Worse still, Emslie et al. showed that 62% of depressed adolescents treated with psychotropic medications experienced a relapse within 78 weeks, despite ongoing treatment (16). Similarly, the Treatment for Adolescents with Depression Study (TADS) found that 47% of remitted patients and 67% of non-responders reported depression recurrence (15). Yet, despite concerted and increasingly sophisticated research in recent decades, early biomarkers to reliably predict adolescent depression trajectory have remained elusive.

### Shortcomings of the Categorical Diagnostic System

Although progress has been slow in identifying definite biobehavioral predictors of depression outcomes in youth, mounting evidence has highlighted the prevailing categorical diagnostic framework itself as a major impediment to understanding the neurobiology of depression and other psychiatric conditions. At issue are the heterogeneous and largely arbitrary criteria used to define clusters of symptoms as particular psychiatric diagnoses in the Diagnostic and Statistical Manual of Mental Disorders (DSM) (17) and similar diagnostic schema such as the International Statistical Classification of Diseases and Related Health Problems (ICD) (18). As the presence and severity of specific symptoms varies widely among psychiatric patients as well as the general population (see section Shortcomings of the Categorical Diagnostic System below), the DSM relies on severity “cut-offs” and endorsement of minimum numbers of super-threshold symptoms to achieve definitive diagnoses. Though convenient for clinicians, the resulting labels obscure crucial inter-individual differences by collapsing numerous disparate clinical presentations into a single putative disorder (19).

In the case of major depressive disorder, current DSM-5 guidelines identify nine core symptoms (Table 1), many of which encompass multiple further variations. Adults who experience at least five of these nine symptoms for a sufficient period (most of the day, nearly every day for a minimum of 2 weeks) meet criteria for a major depressive episode (17). Under this paradigm, a total of 227 possible symptom combinations fulfill the clinical definition of “major depressive disorder,” and it is possible for people with no overlapping symptoms to nevertheless share the same depression diagnosis (20). Nor is this merely a hypothetical concern; a recent survey of psychiatric symptomatology among 1,500 depressed adults identified 170 distinct symptom combinations fulfilling diagnostic criteria for major depression, with variable rates of occurrence (20).

**Table 1:** DSM-5 diagnostic criteria for major depressive disorder.

**#**	**Symptom criteria (Any 5+ for diagnosis)**
1	Depressed mood
1^a^	OR irritability in youth
2	Anhedonia OR loss of interest
3	Significant weight change OR appetite disturbance (increase OR decrease)
3^a^	OR failure to achieve expected weight gains in youth
4	Sleep disturbance (hypersomnia OR insomnia)
5	Psychomotor disturbance (agitation OR retardation)
6	Fatigue OR loss of energy
7	Feelings of worthlessness OR excessive guilt
8	Diminished ability to concentrate
9	Recurrent thoughts of death OR suicidal ideation OR a suicide attempt

a *May substitute alternative criteria in children and adolescents*.

The DSM-5 criteria for diagnosing major depressive disorder in children and adolescents include all the symptoms listed for adults in Table 1 as well as two possible alternatives: irritable mood may be substituted for depressed mood (item 1), and failure to achieve expected weight gains may be substituted for significant weight changes or appetite disturbances (item 3) (17). In other words, diagnostic criteria for depression are even less consistent in youth than they are in adults. Additional factors that may drive variability in pediatric depression diagnoses include: (a) potential difficulty identifying or reporting the type, intensity, or chronology of symptoms for young patients (21); (b) the internalized nature of several symptoms limiting insights from informants such as parents or teachers, often resulting in contradictory information (22); (c) symptoms of major depressive episodes potentially varying over time, settings, or developmental stages (23); and (d) the confounding effects of common comorbidities such as anxiety and substance use (24).

Accordingly, reliable diagnosis of adolescent depression requires experienced clinicians to accurately discern the presence and severity of numerous symptoms, synthesize potentially conflicting reports from patients and informants, and verify or revise their conclusions through sustained longitudinal observation. These involved procedures represent a serious burden on limited community mental health resources, can delay access to potentially time-sensitive interventions, and perpetuate reductive illness classifications with limited neurobiological validity.

### Better Understanding of Adolescent Depression via Dimensional Approaches

In response to these challenges, our group and a growing number of others have shifted toward a dimensional investigative approach that focuses on continuous measures of specific psychiatric symptoms rather than binary classification into broad diagnoses. The dimensional approach views psychiatric symptoms as narrowly defined clinical manifestations of underlying neurobiological abnormalities that may be shared across multiple categorically defined disorders (e.g., anhedonia is a hallmark of both depression and schizophrenia). Whereas, studies centered around categorical diagnoses treat symptom heterogeneity and comorbidity as limitations to be overcome, the dimensional framework treats inter-individual variability as a strength to be leveraged (25). Symptom severity can be readily quantified through standard clinician-rated and self-reported assessments, including many specifically designed for use with adolescents (23, 26). Additionally, the straightforward conceptual mapping of many symptoms onto core functional domains (see section Anhedonia as a Clinical Manifestation of Reward Dysfunction) facilitates translation of insights from animal models and *vice versa*. Another key facet of dimensional research is recruitment of clinically diverse cohorts in order to capture the full range of symptom severity. In marked contrast to the restrictive inclusion criteria used in categorical research to limit variability, dimensional investigations aim to enroll individuals with a variety of psychiatric conditions, including comorbid and sub-threshold symptomatology, resulting in more representative samples and more generalizable findings (27). In recognition of these advantages, the National Institute of Mental Health (NIMH) has promulgated the Research Domain Criteria (RDoC), a set of guidelines designed to promote the adoption of dimensional investigative methods and provide a unified framework for interpreting results (28, 29). The RDoC calls for researchers to focus on identifying specific, biologically meaningful indices of psychiatric phenomena in order to identify shared and distinct etiological mechanisms and inform the next generation of targeted interventions.

Here, we provide a targeted review of these developing trends in research methodology and their application to the study of reward dysfunction in adolescent depression and beyond. We discuss the emergence of the dimensional investigative approach over the past decade, as exemplified by our group's evolving corpus of work as well as a selection of landmark studies by other researchers. Particularly, we aim to demonstrate how a focus on anhedonia has enabled us to leverage multimodal neuroimaging and biological measures and identify shared mechanisms of reward dysfunction across youth with diverse clinical profiles, including depression but also anxiety and behavioral issues. Section Reward Dysfunction as a Promising Predictor of Adolescent Depression Trajectory highlights anhedonia as a direct reflection of deficits in reward processing and an ideal candidate for the dimensional approach advocated in this review. Section Neuroimaging Reward Dysfunction in Adolescent Depression discusses results from our comprehensive adolescent neuroimaging program, including task-based functional magnetic resonance imaging (fMRI) to probe reward processes directly, resting-state fMRI to study key subcortical reward circuitry as well as whole-brain networks, proton magnetic resonance spectroscopy (^1^H MRS) to investigate potential neurochemical alterations within the reward system, and diffusion-weighted imaging (DWI) to examine white matter integrity in reward-related tracts. Section Immune Function and Inflammation in Depression and Reward Dysfunction presents findings from our studies of immunological and inflammatory biomarkers using detailed assays of peripheral cytokines and kynurenine pathway metabolites in youth. Throughout, we emphasize how integration of multiple research modalities through data-driven techniques can further enhance understanding of adolescent depression and advance the goals of identifying objective outcome predictors and targets for personalized early interventions.

## Reward Dysfunction as a Promising Predictor of Adolescent Depression Trajectory

Across species, adolescence is often defined as a period of development when reward-seeking behaviors are dominant, attributed to the rapid maturational changes within the corticolimbic reward circuitry (30, 31). At the same time, adolescence is a critical period when symptoms of many prodromal psychiatric conditions, including depression, anxiety, substance abuse, and psychosis, first emerge (30–33). This increased incidence has been attributed to rapid changes in the brain during adolescence, involving synaptic pruning, myelination, neurotransmission, and the formation of intrinsic functional circuits found in the adult brain (32, 34). As such, deviations from the typical maturation of reward circuitry may underlie the emergence of depressive symptomatology in youth regardless of categorical diagnosis (35, 36). At the same time, the increased neuroplasticity of reward circuits during adolescence provides a unique developmental window for preventive therapies and early interventions targeting reward dysfunction. It is therefore crucial to identify such pathophysiological processes early, ideally before they progress to a full-blown depression episode, in order to limit the effects of chronicity and development of maladaptive behaviors.

### Anhedonia as a Clinical Manifestation of Reward Dysfunction

Anhedonia, defined as the reduced capacity to experience pleasure, is a salient clinical feature of several psychiatric disorders but plays an especially prominent role in depression, where it is considered a core diagnostic symptom (see Table 1) (37). Several features make anhedonia an appealing target for a dimensional investigative approach. Clinically, anhedonic patients may report reduced motivation to pursue potentially pleasurable activities (anticipatory anhedonia), reduced experience of pleasure during such activities (consummatory anhedonia), or both. As such, anhedonia appears to directly reflect deficits in reward function, particularly the key processes of reward anticipation and reward attainment (38, 39). In adolescents, anhedonia often presents as a prodromal symptom prior to the onset of well-defined psychopathology (40, 41) and is associated with less favorable treatment responses (42–44), including persistence of depression symptoms into adulthood (8). Importantly, anhedonia is also linked to increased suicide risk in both pediatric and adult populations (45, 46).

Anhedonia can be readily quantified using a variety of questionnaires, including the 14-item Snaith-Hamilton Pleasure Scale (SHAPS) (47) and the more detailed Temporal Experience of Pleasure Scale (TEPS), which includes 10 items related to anticipatory anhedonia and 8 items related to consummatory anhedonia (48). Though originally developed with adult populations in mind, both scales have subsequently been evaluated for use in adolescent samples. A 2006 validation study of the SHAPS in a cohort of 585 high-school freshmen (age ~14.5) reported high construct validity and supported use of the SHAPS in adolescent populations (49). While the TEPS has not been explicitly validated in adolescents, both anticipatory and consummatory TEPS subscales were found to have high internal consistency (ordinal α > 0.8) in a sample of 449 adolescents (age 13–19) in a recent evaluation of an unrelated social anhedonia questionnaire that included the TEPS for external validation (50). Additionally, a series of studies in university undergraduates reported high measurement and structural invariance for the TEPS across time, gender, and culture (51, 52), particularly when anticipatory and consummatory anhedonia were further subdivided into abstract and contextual components [but see (53)].

### Anhedonia Predicts Worse Adolescent Depression Outcomes

**Figure 1:**
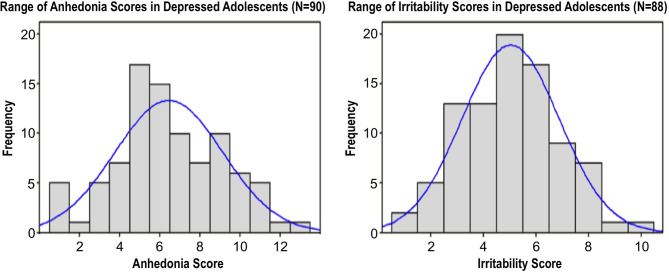
Distribution of anhedonia scores (derived from 1 CDRS-R and 2 BDI questions, range: 1-13) and irritability scores (derived from 1 CDRS-R and 1 BDI question, range: 1-10) in a sample of 90 depressed adolescents. Histograms superimposed with corresponding normal distributions. Adapted from (54).

Several studies have now concluded that anhedonia accounts for a significant proportion of the variance in adolescent depression outcomes. A large cross-sectional analysis by our group examined clinical correlates of two core symptoms, anhedonia and irritability, in a cohort of 90 adolescents (ages 12–20, M ± SD 16.4 ± 2.2 years, 51 female) (54). All participants presented with a current depressive episode of ≥6 weeks duration and moderate or greater severity, defined as a score of ≥35 on the Children's Depression Rating Scale-Revised (CDRS-R) (55), a standard clinician-rated scale to assess depression severity in youth. As illustrated in Figure 1, both anhedonia and irritability exhibited broad dimensional variability with approximately normal distributions across subjects, underscoring the high degree of variation in clinical presentation even among youth diagnosed with a significant current depressive episode.

**Figure 2:**
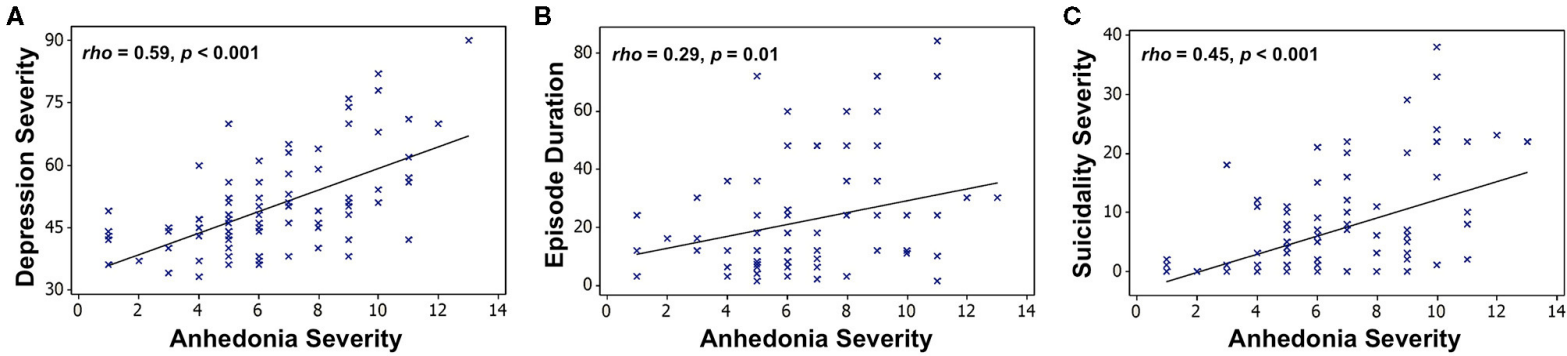
Associations between anhedonia severity and **(A)** overall depression severity (CDRS-R minus the anhedonia question), **(B)** suicidality (Beck Scale for Suicidal Ideation), and **(C)** episode duration (months) in a sample of 90 depressed adolescents. Scatterplots superimposed with Spearman correlation lines of best fit. Adapted from (54).

However, we found that only anhedonia, not irritability, was associated with poorer clinical outcomes, including greater overall illness severity, longer episode duration, and increased suicidality (Figure 2), as well as a larger number of depressive episodes (54).

**Figure 3:**
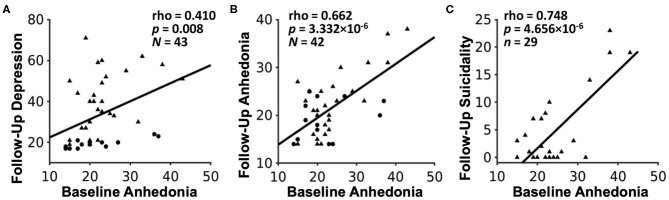
Associations between baseline anhedonia severity and future **(A)** depression severity, **(B)** anhedonia severity, and **(C)** suicidality, assessed after ~24 months in a sample of 29 adolescents with mood and anxiety symptoms (triangles) and 14 healthy adolescent controls (circles). Plots show Pearson partial correlations controlled for baseline age and sex. Adapted from (56).

Moreover, we recently reported initial results from a follow-up study examining longitudinal outcomes after ~2 years in a mixed group of 29 adolescents with mood and anxiety symptoms as well as 14 healthy controls (56). In line with our earlier results, we found that only anhedonia, but not irritability, at baseline was associated with more severe clinical prognosis, including future depression severity, anhedonia severity, and suicidality (Figure 3). This study also illustrates a key principle of our dimensional analysis approach, namely that symptom severity lies along a continuum from typical (low severity) to potentially pathological (high severity) even among nominally healthy individuals, denoted by circles in Figure 3. Additional findings from this study relating fMRI measures of reward function to future clinical outcomes are detailed in section Reward Function Predicts Adolescent Depression Outcomes below.

Several independent lines of evidence further support our conclusion that anhedonia, as an index of reward dysfunction, plays a uniquely predictive role in the trajectory of depression. A multivariate analysis of 334 depressed youth in the Treatment of Resistant Depression in Adolescents (TORDIA) study found that, out of five symptom dimensions, only anhedonia predicted longer remission time and fewer depression-free days over 24 weeks; findings remained significant even when accounting for overall depression severity (57). A study of 75 depressed adult psychiatric inpatients similarly found that anhedonia predicted treatment non-response among depressed inpatients (58), while our study of 135 adults hospitalized for acute suicide attempt or high suicide risk documented that anhedonia as well as entrapment were independently associated with suicidality scores (59).

Taken together, these findings support the notion that anhedonia reflects key aspects of reward dysfunction across ages and may map particularly well onto underlying neural processes that contribute to the development and maintenance of depression in youth.

### Linking Reward Function to Reward Circuitry

Reward function is inherently complex, involving multiple discrete processes and phases, including reward anticipation, attainment, and valuation (60, 61). Neuroanatomically, however, many reward processes engage a common circuit linking regions of the dopaminergic midbrain, basal ganglia, and frontal cortex. In animals ranging from rodents to non-human primates, the processes of reward anticipation and, particularly for novel stimuli, reward attainment rapidly engage midbrain populations of dopaminergic neurons in the ventral tegmental area and substantia nigra (62–65). These reward-encoding signals propagate via ascending projections to the ventral striatum (mesolimbic pathway) and dorsal striatum (nigrostriatal pathway) *en route* to cortical targets, including the ventromedial prefrontal cortex (PFC), subgenual and pregenual anterior cingulate cortex (ACC), and supplementary motor area (39, 66–80). As will be discussed further in section Neuroimaging Reward Dysfunction in Adolescent Depression, a substantial body of literature now ties the clinical phenomenology of anhedonia to disturbances in this core reward circuit (81–84). At the same time, neuroimaging studies of anhedonia and related constructs frequently report abnormalities beyond the canonical reward circuitry, highlighting the need for an integrative, empirically driven approach to fully characterize the interdependent neural processes supporting reward function.

## Neuroimaging Reward Dysfunction in Adolescent Depression

Non-invasively studying the human brain *in vivo* is a major technical challenge. Nevertheless, a robust research community has emerged around the study of adolescent depression using a variety of increasingly sophisticated neuroimaging techniques. Since no single technique can adequately capture all aspects of neural anatomy and function, combining evidence from complementary modalities is crucial for verifying and clarifying results. For example, several longitudinal studies using functional magnetic resonance imaging (fMRI) have found that altered neural activation profiles during reward anticipation and attainment in non-depressed adolescents predicted the onset of future depression (85, 86). While these fMRI studies suggest that detectable changes in reward-related neural activity precede the onset of adolescent depression symptoms, this conclusion is strengthened considerably by corroborating evidence from electroencephalography (EEG), a completely different neuroimaging technique. In an EEG study of 444 healthy female adolescents (age 13–15), blunted reward positivity and reduced delta band amplitude during reward attainment predicted the onset of depressive disorders and greater depression severity at 18-months follow-up, even when controlling for baseline depressive symptoms, lifetime psychiatric history, and parental psychiatric history (87, 88). By bringing together multimodal results from both fMRI and EEG studies, a recent meta-analysis was able to identify a set of reward system alterations that consistently preceded the onset of adolescent depression symptomatology (89). However, studies employing multiple neuroimaging techniques within the same cohort are rare.

In this section, we review findings in adolescent depression based on a variety of imaging methods, focusing particularly on advanced MRI techniques employed by our group to examine the neural underpinnings of reward dysfunction. These include task-based and resting-state fMRI to assay neural activity across the brain, proton magnetic resonance spectroscopy (^1^H MRS) to detect neurochemical concentrations in targeted regions, and diffusion-weighted imaging (DWI) to model structural connectivity in white matter. As we seek to demonstrate, combining data from multiple neuroimaging modalities, as well as other biological measures (see section Immune Function and Inflammation in Depression and Reward Dysfunction), within a single cohort substantially enhances our ability to make inferences about brain function and its role in the emergence of psychiatric illness.

### High-Resolution Neuroimaging and High-Fidelity Analysis Approach

Functional MRI is a powerful, though indirect, method to measure neural activity. Through a phenomenon known as neurovascular coupling, localized increases in neural activity trigger increased perfusion with oxygen-rich blood to meet metabolic demands (90). Thanks to the magnetic properties of hemoglobin, these shifts in blood flow give rise to an endogenous contrast mechanism known as the blood-oxygenation-level-dependent (BOLD) signal, which can be detected and tracked over time using rapid, inhomogeneity-sensitive fMRI sequences (90–92). The combination of relatively high spatial fidelity, whole-brain coverage, and increasingly rapid sampling rates through the use of accelerated imaging techniques make fMRI an attractive method for studying human cognition in healthy and diseased states. Over the past two decades, the body of basic and clinical neuroscience literature utilizing fMRI has grown exponentially.

Despite these efforts, considerable uncertainty remains regarding *in vivo* reward function, particularly in youth. Much of the difficulty in mapping reward circuitry can be attributed to limitations in MRI methodology. This is particularly true for subcortical structures such as the ventral tegmental area (source of dopaminergic reward signals) and the habenula (inhibits dopamine signals in response to negative outcomes). These and other key reward-related structures have limited anatomical tissue contrast and have proven difficult to reliably segment, with the most accepted methods rely on heuristic anatomical landmarks (93–96). These issues are compounded in fMRI studies, where the combination of low spatial resolution, signal contamination from poorly delineated tissue boundaries, and high physiological noise make subcortical reward structures especially challenging to study (93, 97).

Our laboratory has therefore pursued a high-resolution imaging acquisition and processing approach modeled after the Human Connectome Project (HCP) (98) and designed to systematically address these limitations. As early adopters of multiband acceleration, we have now collected hundreds of whole-brain fMRI datasets at 3T with high spatial resolution (2.3 mm isotropic vs. ≥3 mm isotropic for traditional sequences) and sampling rates (repetition time = 1 s vs. ≥ 2 s for traditional sequences). These high-resolution fMRI sequences allow for substantially improved signal localization and enable us to fully capitalize on advanced tools such as the HCP Pipelines to perform sophisticated preprocessing procedures (99), ICA-FIX to automatically classify and remove structured noise (100–102), and multimodal surface matching (MSMAll) to robustly align corresponding cortical areas and maximize spatial fidelity (103–106). Wherever possible, our analyses employ threshold-free cluster enhancement (TFCE) for adaptive, data-driven identification of meaningful fMRI effects (107) in conjunction with non-parametric permutation-based statistics for precise control of family-wise error (FWE) rates (108, 109) with minimal assumptions about data distribution (109–111), in line with current best practices. Finally, we ascribe to a transparent “open science” philosophy and have endeavored to make study preprints, final results datasets, and analysis code freely and readily available to the community. While our neuroimaging procedures have evolved over the past years and will continue to do so as improved techniques become available, we emphasize the importance of a holistic approach to high-fidelity neuroimaging at each stage of acquisition and processing to study neural function as accurately and reproducibly as possible.

### Task-Based Functional Magnetic Resonance Imaging

A major use-case for fMRI is to examine neural activation during specific activities or mental processes. In task-based fMRI, participants can complete a wide range of behavioral task paradigms while being scanned to capture corresponding neural activity, including reward processing. One of the earliest and most prominent researchers to use fMRI tasks to specifically examine adolescent reward processing and associated deficits was Erika Forbes (112). Using a probabilistic reward task, Forbes and colleagues documented reduced activation in the bilateral striatum and portions of the orbitofrontal cortex (OFC), key reward processing regions, during reward anticipation and reward attainment in 14 adolescents/pre-adolescents (ages 8–17) with major depressive disorder vs. 17 age-matched healthy controls (72). The finding of reduced striatal reward response was corroborated in a follow-up study with an expanded sample of 15 depressed adolescents/pre-adolescents and 28 healthy controls, which also reported that individuals with less caudate activation had lower overall positive affect, assessed by telephone outside the laboratory setting (76). In subsequent research, Forbes et al. found that the degree of striatal activation to rewards positively correlated with positive affect while medial PFC activation positively correlated with depression symptomatology in a large sample of young people with no history of psychiatric illness (*N* = 77, ages 11–13) (113). Crucially, they also found evidence that striatal reward reactivity predicted subsequent improvement in mood and anxiety symptoms following 8 weeks of open-label treatment (behavioral or behavioral + antidepressant therapy) in a sample of adolescents (ages 10–16) with major depression (114). As detailed below, our group has likewise made extensive use of task-based fMRI to probe key aspects of reward function and their relevance to adolescent mood and anxiety disorders.

#### The Reward Flanker Task

As discussed in section Reward Dysfunction as a Promising Predictor of Adolescent Depression Trajectory, reward dysfunction and anhedonia in adolescent depression entail deficits in multiple reward processes. For example, some patients may lack the motivation to pursue potentially pleasurable activities (i.e., deficits in reward anticipation), while others may seek out such activities but find that they experience little or no pleasure as a result (i.e., deficits in reward attainment). To better delineate neural activity during these distinct phases of reward processing, our group has developed the Reward Flanker Task (RFT). Using the RFT, we are able to directly examine neural responses to reward anticipation (monetary cues) and reward attainment (monetary outcomes), but also a number of important additional constructs related to outcome uncertainty and reward prediction errors.

Implementation of the RFT is described extensively in our previous publication (115). Briefly, the RFT combines elements of the earlier Incentive Flanker (116) and Monetary Incentive Delay (117) tasks, both of which are widely used in psychological as well as neuroimaging research to examine reward responses. Trials in the RFT consist of:

1) Cue indicating high (50%), low (10%), no (0%), or unknown (?) trial type for 4–6 s

2) Flanker stimulus (short string with a unique letter, e.g., CCHCC) for 300 ms

3) Calibrated response window with a blank display for ≤ 1,700 ms

4) Feedback indicating high (50%), low (10%), or no (0%) reward earned for 2 s

5) Inter-trial-interval with a blank display for 4–6 s.

Unknown cues yield high, low, and no reward outcomes equally. During the response window, participants attempt to identify the unique target letter from the flanker stimulus using button presses. To keep the task challenging and ensure that some errors are made, the amount of time a participant is allotted to make a response is set at 0.8–1.5 times their mean reaction time during a pre-scan practice session. Correct responses to flanker stimuli result in winning a high, low, or no reward outcome based on the trial type. A total of 120 trials (30 of each type) are presented in pseudo-random order across four 4-min fMRI runs.

Compared to other reward paradigms such as the Incentive Flanker and Monetary Incentive Delay, the RFT has several novel features. First, cues used to signal the reward value of each trial are presented with varying durations and for sufficient lengths to allow for the separation of brain activity during reward expectancy from attainment despite the slow hemodynamic response. Second, the task uses three levels of reward value (high, low, and no reward) to examine brain activity in response to increasing reward value for both expectancy and attainment. Third, the task includes unknown reward cues, allow us to dissociate responses to certain vs. uncertain reward outcomes. In our studies, the contrast of reward attainment following uncertain vs. certain cues is referred to as positive prediction error. Finally, as cues elicit reward anticipation such that a specific, effortful response is required to obtain the reward, rather than passive anticipation of a definite reward, the RFT is particularly well-suited to probe motivational deficits in anhedonia.

**Figure 4:**
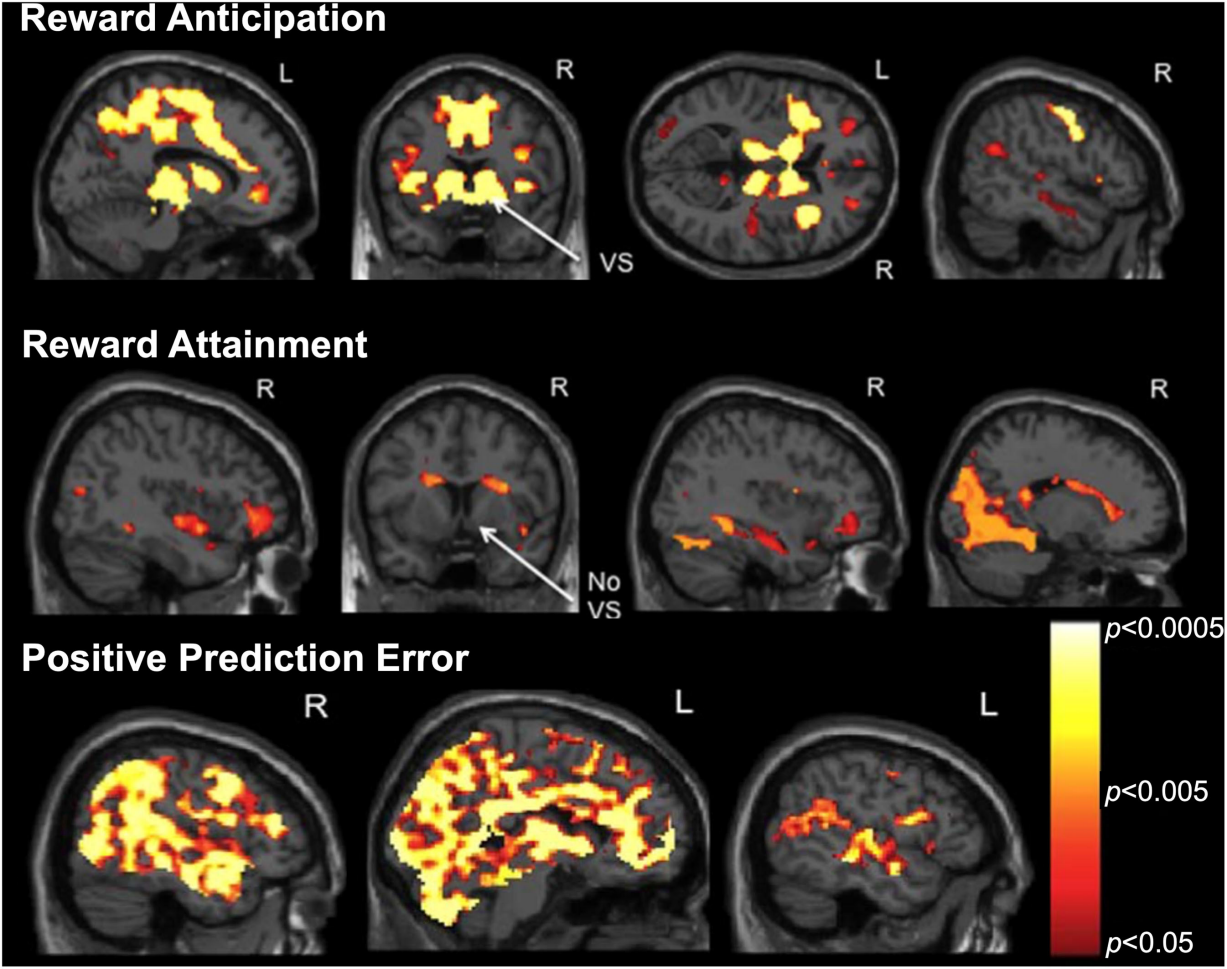
Reward Flanker Task activation maps (*p*_*TFCE*−*FWE*_ < 0.05) from a pilot study of 22 adolescents (16 depressed, six healthy controls) showing neural responses to (**top**) reward anticipation, (**middle**) reward attainment, and (**bottom**) positive prediction error contrasts. Note the differential engagement of ventral striatum (VS) during reward anticipation but not attainment. Adapted from (115).

Following the development of the RFT, we conducted a pilot study to examine brain activation during distinct reward processes in a sample of 22 psychotropic medication-free adolescents, including 16 with primarily mood and anxiety symptoms and 6 healthy controls (115). Results from our whole-brain analyses, controlled for false discovery rate (FDR), are displayed in Figure 4 (all *p*_*TFCE*−*FDR*_ < 0.05). We found that reward anticipation activated a large bilateral network including default-mode, salience, and limbic regions important for reward appraisal and conflict resolution. Reward attainment engaged a more restricted network including the hippocampus, medial temporal lobe, dorsal caudate, and occipital areas related to memory and emotion processing but notably not the ventral striatum. Additionally, we found that positive prediction errors yielded widespread cortical activation in many of the same regions identified in during reward anticipation/attainment, including the ventral striatum. Exploratory analyses at a relaxed threshold (uncorrected *p* < 0.001) further identified several positive correlations between anhedonia severity and RFT activation, specifically in the right angular gyrus during reward anticipation and in the left precuneus during positive prediction errors. These results support the ability to differentiate reward processes and identify links to clinical reward dysfunction in adolescents using the RFT. A follow-up RFT study in an expanded adolescent cohort is currently in preparation, with findings largely corroborating our initial pilot results; additionally, three reward networks derived from this follow-up study served as the basis for our recent graph theory analysis using resting-state fMRI, described in section Using Graph Theory to Bridge Resting-State and Task-Based fMRI Studies below.

#### Reward Function Predicts Adolescent Depression Outcomes

**Figure 5:**
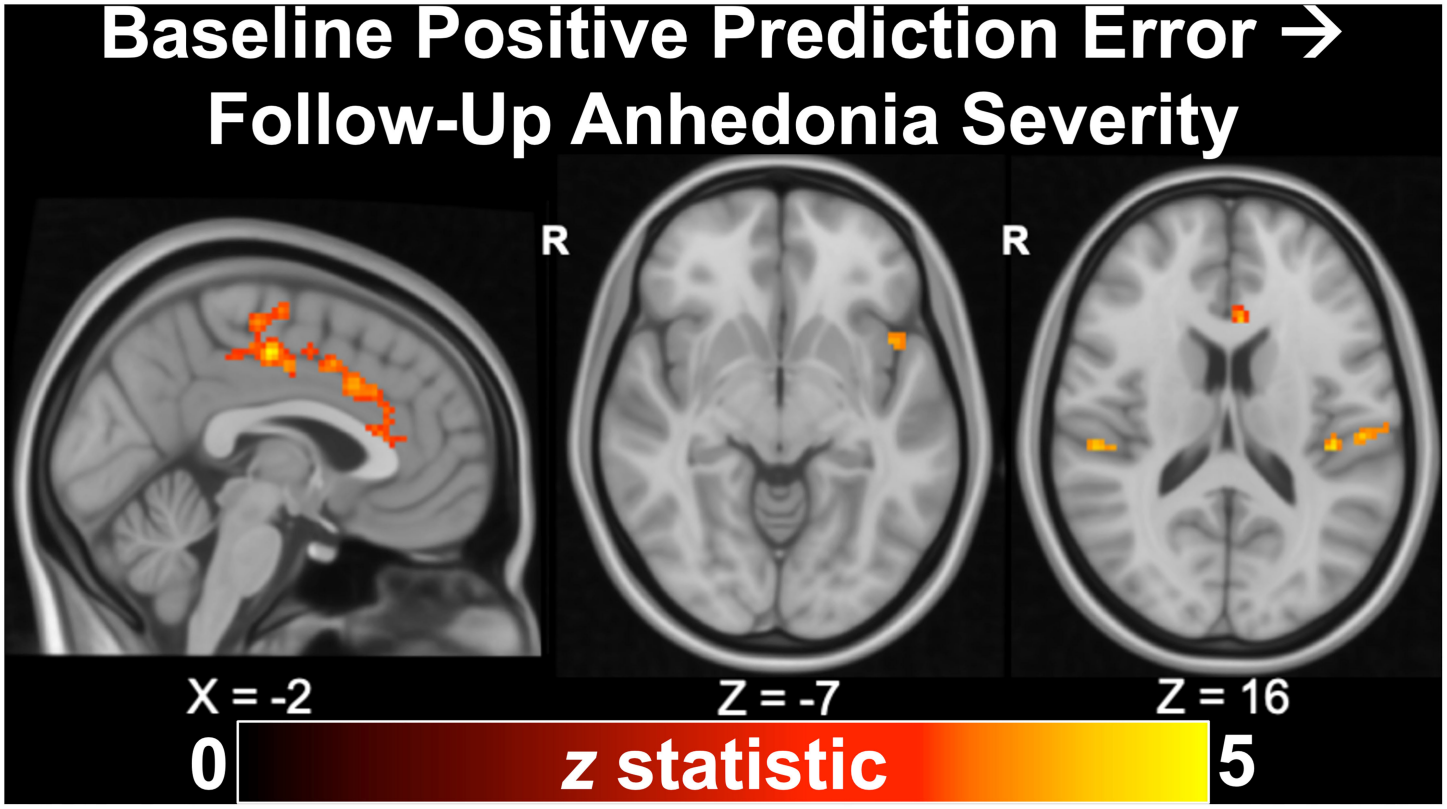
Activation to positive prediction errors in the Reward Flanker Task predicted clinical outcomes at ~24 months follow-up, including anhedonia severity (*p*_*TFCE*−*FWE*_ < 0.05), in a longitudinal study of 32 adolescents (22 with mood/anxiety symptoms, 10 healthy controls). Excerpted from (56).

As described previously in section Task-Based Functional Magnetic Resonance Imaging, we recently published findings from a longitudinal study of 43 adolescents indicating that anhedonia, but not irritability, predicted depression severity and suicidal ideation at ~24 months (56), corroborating our earlier cross-sectional results (54). In additional to clinical associations, we examined whether measures of neural reward function at baseline would predict future clinical outcomes in a subset of 22 depressed and 10 healthy adolescents with RFT fMRI data. In the full sample, we found that activation during positive prediction error in the dorsal ACC, mid-cingulate, anterior insula, operculum, and motor cortex significantly (all *p*_*TFCE*−*FWE*_ < 0.05) predicted future anhedonia severity (Figure 5). Activation during positive prediction error in the left angular gyrus further predicted future depression severity in the full sample. In the depressed-only cohort, analyses indicated that positive predication error activation in the dorsal ACC/mid-cingulate again predicted future anhedonia, while activation in the medial precuneus/posterior cingulate during reward attainment predicted future suicidality (not assessed in controls due to minimal range). Thus, altered activation during reward processing, specifically in cingulate areas related to evaluating reward outcomes as well as regions important to pain and salience processing, was linked to future reward deficits, indexed by anhedonia.

#### Altered Emotion Processing and Self Perception in Adolescent Depression

While the majority of our work has used monetary rewards to probe reward circuitry, as in the RFT, happy facial expressions are also commonly used as rewarding social stimulus in studies of adolescent reward processing (112). Studies have also consistently reported emotion processing deficits in people with depression, who are less accurate and more negative in their interpretation of facial affect than healthy individuals (118). This pattern is reflected in neuroimaging data, with depressed adults showing stronger responses to sad/angry faces and weaker activation to happy faces in the striatum and amygdala relative to healthy controls (119). Notably, a study of children at high risk for developing depression (*N* = 38) yielded largely consistent findings of amygdala hyperactivation to fearful faces and ACC hypoactivation to happy faces relative to low-risk controls (*N* = 23) (120). Using a similar approach, we sought to examine neural activation during emotional valence judgements in depressed adolescents (121). A total of 19 psychotropic-medication-free depressed and 18 healthy control adolescents completed a 3T fMRI task where they were shown faces with neutral, happy, sad, or fearful expressions and were asked to rate the sadness of the expressions. Analyses indicated that depressed adolescents relative to controls had reduced activation in the left superior temporal gyrus while judging sad and neutral faces. Within the depressed group, greater illness severity was associated with stronger neural activation in major reward and aversion processing regions while judging sad faces (e.g., putamen, ventromedial PFC, amygdala, anterior insula) and fearful faces (e.g., dorsal ACC, anterior insula). Anhedonia scores in the depressed group showed similar positive correlations with activation while judging sad faces (e.g., putamen, caudate, and amygdala) but were uniquely anticorrelated with activation while judging happy faces (e.g., dorsal ACC, anterior insula).

While evidence supports a negative bias when evaluating the emotional valence of others, excessively negative self-perception is even more characteristic of depression. We therefore conducted a separate 3T fMRI study of self-referential word processing in a group of 23 depressed and 18 healthy control adolescents (122). Subjects were presented with words describing positive and negative personal traits and were asked to either judge whether the trait: (1) applied to themselves, or (2) was a good trait to have in general. Adolescents with depression endorsed more negative traits but exhibited stronger activation in the posterior cingulate while making positive self-judgements than their healthy counterparts. Within the depressed group, positive self-perception was negatively correlated with multiple dimensional measures of symptom severity, including self- and clinician-rated depression severity, social and general anxiety, irritability, and anhedonia. Additionally, a psychophysiological interaction analysis of task-evoked functional connectivity among four cortical midline regions of interest revealed diminished connectivity of the dorsomedial PFC—the same region identified in our earlier study of striatal resting-state connectivity in adolescent depression (section Resting-State Functional Connectivity of the Striatum in Adolescent Depression)—during self-judgement.

Naturally, these studies should be interpreted with caution given their small sample sizes. Nevertheless, they offer insight into the perception of self and others in adolescent depression, suggesting that neural reward circuitry and clinical reward deficits can influence seemingly distinct cognitive processes related to internally and externally directed judgements. Interestingly, a recent study in a larger sample of depressed and healthy adolescents (*N* = 120) combined these approached by having subjects identify their own emotional expressions from photos taken 1–2 weeks prior to the task. Results indicated that, unlike when processing the emotional expressions of others, recognition accuracy among depressed adolescents was highest for happy faces. Analysis of fMRI data further revealed a that elevated amygdala connectivity with regions of the ACC and PFC during the task was associated with increased suicidality and was highest in youth with a history of suicide attempts (123).

### Resting-State Functional Magnetic Resonance Imaging

While task-based fMRI seeks to identify brain activity in response to external stimuli, resting-state fMRI is a complementary approach that provides unique information on brain function, particularly its organization into large-scale networks. In resting-state fMRI, BOLD signals are measured in the absence of external task stimuli, with subjects free to let their minds wander. The most common class of resting-state fMRI analyses focus on resting-state functional connectivity (RSFC), which measures correlations in low-frequency BOLD fluctuations (typically ~0.1–0.01 Hz) between difference regions. Networks identified using RSFC can reflect not only known anatomical connectivity between regions but also areas with no direct anatomical links, implying that correlated activity is instead driven by shared function (124–126). Several features make resting-state fMRI well-suited for research: findings can be compared readily across studies and populations, the low cognitive demand allows for participation of individuals who may not be able to complete complex tasks, and many well-characterized networks can be readily identified even at the single-subject level using hypothesis-free techniques such as independent components analysis.

Numerous studies have now documented resting-state network alterations in adolescent depression and related conditions. Brain structures most frequently implicated in these studies include the anterior cingulate cortex, insula, and especially the default-mode network (127–130), which encompasses regions of the medial PFC, posterior cingulate, lateral temporal lobes, and hippocampus that are suppressed during fMRI tasks (131). Dimensional studies further indicate that changes in resting-state network properties are correlated with adolescent depression severity. For example, Roselinde Kaiser, Diego Pizzagalli, and colleagues recently found that co-activation of the default-mode network and the (typically distinct) fronto-insular network during rest was associated with more severe mood symptoms in a sample of 45 depressed adolescents (ages 13–19) (132); a similar pattern of insula/default-mode hyperconnectivity during an emotional memory task was related mood status in over the subsequent 2 weeks (133). Furthermore, evidence from children/early adolescents indicates that RSFC abnormalities arise very early in the course of illness and may precede the emergence of overt clinical symptoms. Susan Whitfield-Gabrieli's group, for instance, reported default-mode network hyperconnectivity with the subgenual ACC and OFC in 27 children (ages 8–14) with a parental history of major depression vs. 16 age-matched controls with no parental depression history (134). A subsequent study based on a community sample of 54 children assessed longitudinally at ages seven and 11 found that reduced RSFC between the subgenual ACC and dorsolateral PFC at baseline was associated with the development of mood and anxiety symptoms at 4-years follow-up. Moreover, this connectivity pattern predicted future depression symptomatology better than a standard parent-rated clinical scale. Increased RSFC between the medial and dorsolateral PFC was separately associated with future attentional symptoms (135).

As detailed below, our group has utilized resting-state fMRI extensively to examine reward circuitry in adolescent depression, both through seed-based RSFC analyses of *a priori* reward areas (sections Resting-State Functional Connectivity of the Striatum in Adolescent Depression and Habenula Functional Connectivity and Subclinical Depression in Young Adults) and data-driven analyses using graph theory (section Using Graph Theory to Bridge Resting-State and Task-Based fMRI Studies).

#### Resting-State Functional Connectivity of the Striatum in Adolescent Depression

**Figure 6:**
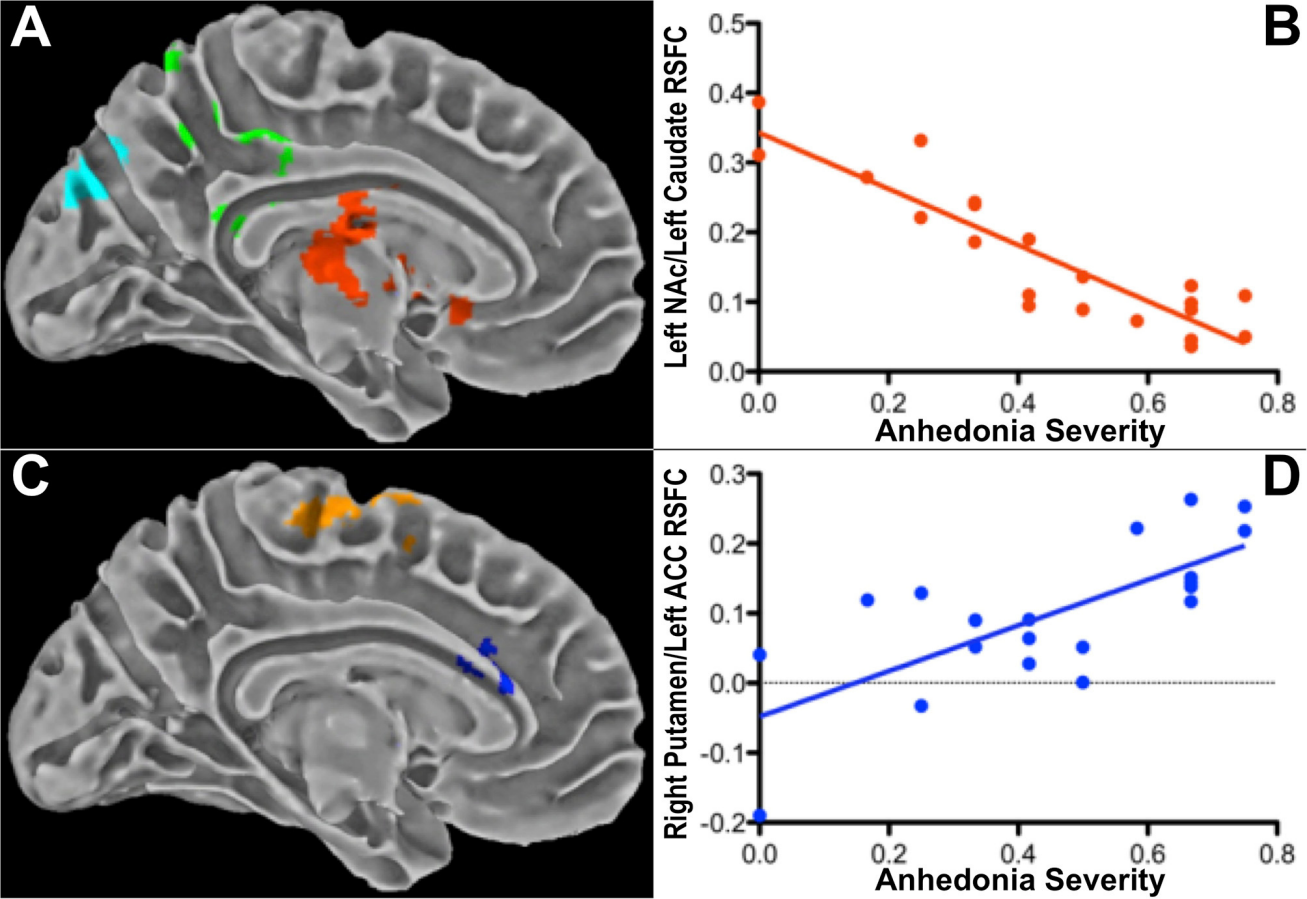
Associations between striatal RSFC and anhedonia severity in 21 depressed adolescents (cluster-level *p*_*Bonferroni*_ < 0.05) after controlling for overall depression severity. Anhedonia correlated **(A,B)** negatively with RSFC between the left nucleus accumbens (NAc) and a cluster (red) containing the left caudate and subgenual ACC; and **(C,D)** positively with RSFC between the right putamen and a cluster (blue) within the dorsal left dorsal ACC. Excerpted from (136).

We conducted an early study of striatum-based RSFC in a cohort of 42 psychotropic-medication-free adolescents, 21 with a current major depressive episode and 21 healthy controls with no history of psychiatric illness (136). Mean timeseries were extracted using spherical seeds (~4 mm radius) from six striatal regions per hemisphere (dorsal caudate, ventral caudate, dorsal caudal putamen, dorsal rostral putamen, ventral rostral putamen, nucleus accumbens) created as part of an earlier study (137). Secondary analyses examined RSFC from three template seeds per hemisphere corresponding to the major divisions of the striatum (caudate, putamen, nucleus accumbens), as defined by the Harvard-Oxford Structural Atlas (138). At the group level, depressed adolescents manifested increased RSFC between all striatal seeds bilaterally and the dorsomedial PFC as well as between the right ventral caudate and the subgenual ACC compared to controls. Controls exhibited stronger RSFC between striatal seeds and extrastriate visual areas in the occipital lobe, between the left dorsal caudate and the superior temporal lobe, and between the ventral caudate and postcentral gyrus. Within the depressed group, overall depression severity was associated with RSFC between the striatum and midline structures including the precuneus, posterior cingulate cortex, and dorsomedial PFC. However, anhedonia severity was associated with distinct striatal RSFC patterns involving the pregenual ACC, subgenual ACC, supplementary motor area, and supramarginal gyrus, even after controlling for depression severity. Symptom correlation results are presented in Figure 6. Although the sample size was modest, these findings provided early evidence for anhedonia as a unique clinical variable capturing facets of reward dysfunction beyond depression severity alone.

#### Habenula Functional Connectivity and Subclinical Depression in Young Adults

While the dopaminergic reward circuit described in section Linking Reward Function to Reward Circuitry is strongly engaged by positive prediction errors (i.e., receipt of unexpected rewards), reward-related dopamine signaling is inhibited by negative prediction errors (i.e., non-receipt of expected rewards) (139). Preclinical work over the past decade indicates that this pattern is driven by a highly conserved circuit centering on the lateral habenula, a small epithalamic nucleus that sends extensive regulatory projections to the dopaminergic midbrain and other monoamine systems (140–143). The habenula responds vigorously to aversive stimuli (144, 145) but is inhibited by rewards (146–148). Further, excitatory habenula stimulation induces depression- and anhedonia-like behaviors in animal models similar to those produced by lesions of the ventral tegmental area and nucleus accumbens (63, 149, 150). As such, the habenula is considered a highly promising candidate structure in the study of clinical reward dysfunction. However, the small size (~30 mm^3^ per hemisphere) and limited anatomical contrast of the habenula with nearby thalamic tissue have severely limited *in vivo* research.

In response to these challenges, our team has worked extensively to refine habenula imaging methodology, co-developing an automated segmentation scheme based on 3T anatomical MRI (151, 152). The technique entails taking ratios of T1-weighted (T1w) to T2-weighted (T2w) images to selectively enhance *in vivo* myelin contrast (153). As the habenula contains more myelin than the surrounding thalamus, this method significantly improves tissue contrast relative to T1w or T2w MRI alone (152). A region-growing algorithm then automatically identifies and assigns probabilistic weights to voxels containing habenula tissue.

**Figure 7:**
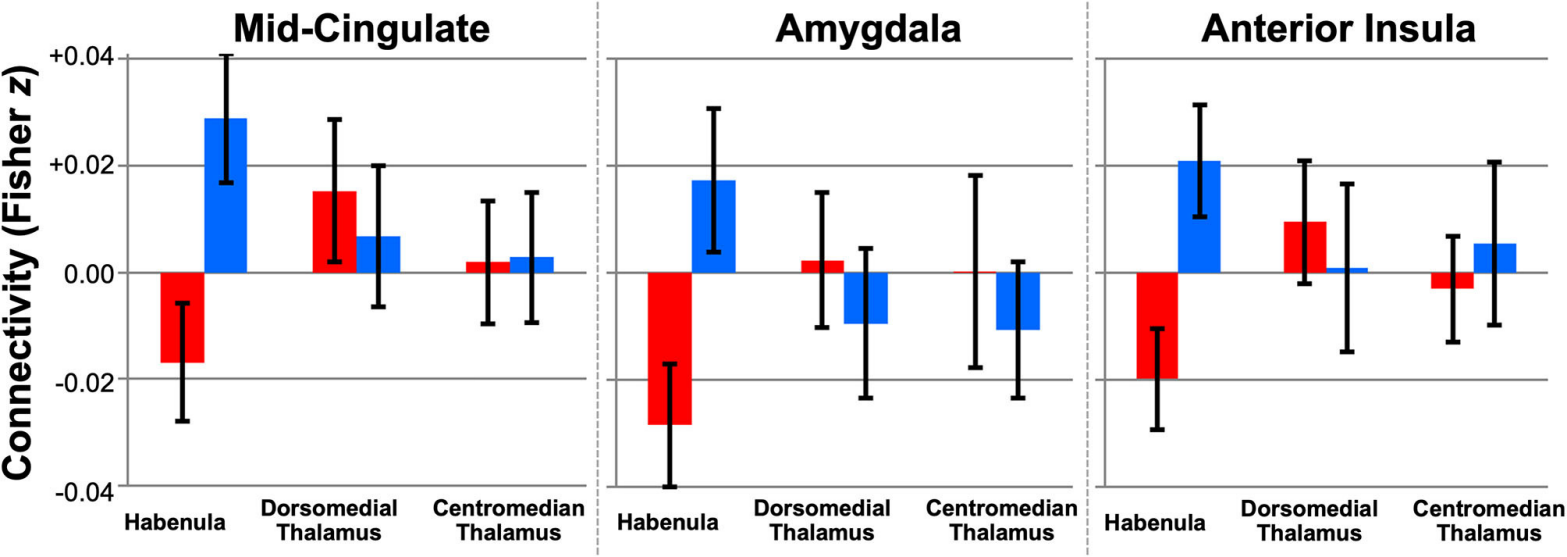
Altered habenula RSFC in young adults from the Human Connectome with high subclinical depression (*N* = 25, red) vs. low subclinical depression (*N* = 25, blue). Plots show seed-to-cluster RSFC values (mean ± 95% CI) for three clusters (mid-cingulate, amygdala, anterior insula) where habenula RSFC differed between groups (exploratory *p*_*uncorrected*_ < 0.001, *k* ≥ 10). No group differences in RSFC were detected for adjacent seeds in the dorsomedial or centromedian thalamus with the same clusters. Excerpted from (154).

Using this technique, we performed the first whole-brain study of human habenula RSFC using high-resolution fMRI data from 50 young adults (ages 22–35) in the HCP, 25 with low and 25 with high subclinical depression scores (154). In line with animal literature, we found extensive RSFC with reward- and aversion-related areas in the full sample, including the ventral tegmental area, nucleus accumbens, dorsal ACC, and periaqueductal gray (*p*_*TFCE*−*FWE*_ < 0.05, controlled for subclinical depression group). At a relaxed exploratory threshold (uncorrected *p* < 0.001, *k* ≥ 10), group contrasts further revealed reduced habenula RSFC with the mid-cingulate, right amygdala, and right anterior insula, regions implicated in mood disorders and aversion processing, in subjects with high subclinical depression scores (Figure 7).

Building on this work, we developed an optimized method for downsampling anatomical-resolution (~0.8 mm isotropic) habenula segmentations to fMRI resolution (~2 mm isotropic) for use as RSFC seeds. These individual-specific seeds account for tissue probability weights, partial volume effects, and template normalization, significantly improving BOLD sensitivity compared to other seeding methods (155). In a follow-up study also incorporating neuroanatomically accurate surface analysis, we created the most detailed maps to date of habenula RSFC in a representative sample of 68 healthy young adults from the HCP. Findings corroborated our initial results and further revealed positive habenula RSFC with the insula, pregenual ACC, and striatum as well as a pattern of weakly negative RSFC throughout task-negative regions of the default-mode network. A forthcoming manuscript will detail findings using the same sophisticated mapping approach in a large, clinically diverse adolescent sample.

#### Using Graph Theory to Bridge Resting-State and Task-Based fMRI Studies

**Figure 8:**
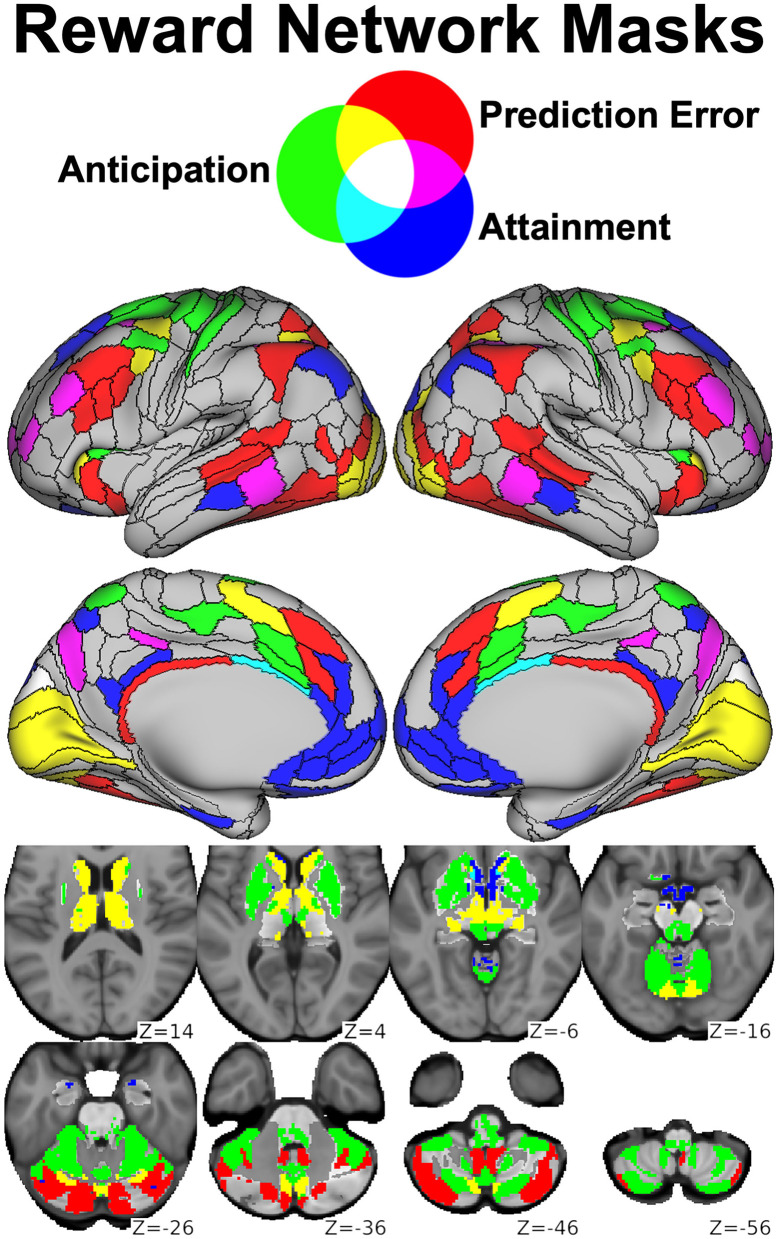
Parcels derived from the Cole-Anticevic Brain-wide Network Partition (black lines) most strongly activated during the RFT by reward anticipation (green), reward attainment (blue), reward prediction errors (red), or some combination thereof (mixed colors) and used as reward networks in our graph theory study. Reprinted from (156).

As described in section The Reward Flanker Task, our task-based fMRI findings using the RFT have identified distinct activation networks engaged by the processes of reward anticipation, reward attainment, and positive prediction error. Building on this work, we conducted a data-driven graph theory analysis to identify resting-state network features associated with psychiatric symptomatology in a sample of 68 clinical adolescents with predominantly mood and anxiety symptoms as well as 19 healthy adolescent controls (156). This analysis capitalized on the Cole-Anticevic Brain-wide Network Partition (157), a recent whole-brain extension of the landmark HCP cortical parcellation (103) that identified 358 additional subcortical parcels on the basis of RSFC patterns. By applying the Cole-Anticevic atlas to task activation maps derived from our updated RFT analysis, we generated network templates corresponding to *Reward Anticipation* (114 nodes), *Reward Attainment* (103 nodes), and *Reward Prediction Error* (117 nodes), reprinted as Figure 8.

Graph theoretical analyses were performed using the Brain Connectivity toolbox (158) to derive three descriptive metrics within the three RFT-derived networks as well as a *Whole Brain* network (750 nodes) derived from the Cole-Anticevic atlas. Strength Centrality represented the sum of edge weights per node and captured the overall influence of each node within the network. Eigenvector Centrality represented the largest magnitude eigenvector per node and captured the influence of each node over highly influential nodes within the network. Local Efficiency represented the inverse shortest path length between each node and its neighborhood and captured how readily information could propagate to other nodes in the network.

**Figure 9:**
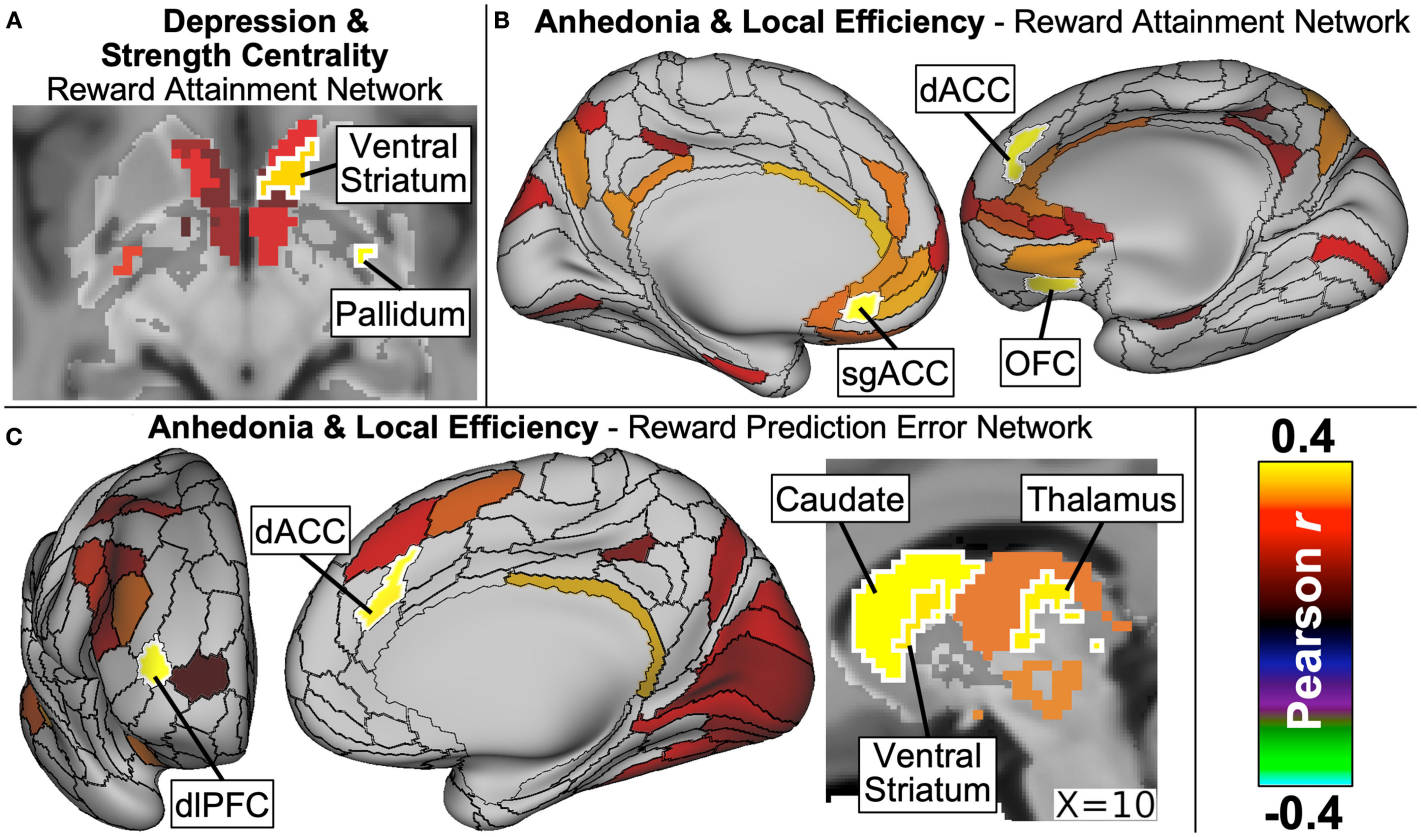
Major findings from our graph theory analysis of resting-state network properties in adolescents with diverse psychiatric profiles. Depression severity **(A)** was positively correlated with strength centrality of ventral striatum and pallidum nodes within the *Reward Attainment* network. Anticipatory anhedonia, meanwhile, correlated with local efficiency **(B)** within the *Reward Attainment* network in the subgenual ACC (sgACC), dorsal ACC (dACC), and OFC, as well as **(C)** within the *Reward Prediction Error* network in the stratum, dACC, and dorsolateral prefrontal cortex (dlPFC). Excerpted from (156).

As detailed in our manuscript now in press (156), dimensional symptom analyses revealed that depression severity significantly correlated with Strength Centrality in two ventral striatum nodes as well as with Strength Centrality and Local Efficiency measures in the right inferior pallidum within the *Reward Attainment* network (Figure 9A). Anticipatory anhedonia, meanwhile, correlated with Local Efficiency in numerous reward-related nodes across networks, including the subgenual ACC within *Whole Brain* and *Reward Attainment* networks, dorsal ACC within *Reward Attainment* and *Reward Prediction Error* networks, OFC within the *Reward Attainment* network, ventral striatum within the *Reward Prediction Error* network, and the dorsal caudate within *Reward Anticipation* and *Reward Prediction Error* networks (Figures 9B,C). Notably, significant correlations were not identified with consummatory anhedonia and were much more limited for total anhedonia (i.e., anticipatory + consummatory scores), highlighting the need for detailed measures of reward dysfunction in dimensional research. Similarly, no significant correlations were identified with anxiety severity, and no significant group differences were detected between clinical and healthy control groups, again underscoring the central role of reward dysfunction in adolescent depression symptomatology.

### Neurochemical Abnormalities Revealed by Magnetic Resonance Spectroscopy

Proton magnetic resonance spectroscopy (^1^H MRS) is a nuclear magnetic resonance technique closely related to MRI that can distinguish protons from various tissue chemicals, allowing for *in vivo* measurement of human biochemistry (159). Localized ^1^H MRS has gained popularity in psychiatric research due to its ability to directly assess the concentrations of many important neurochemical metabolites, including glutamate, glutamine, γ-aminobutyric acid (GABA), N-acetylaspartate, and myoinositol. Of particular interest are GABA and glutamate, respectively, the most common inhibitory and excitatory neurotransmitters in the brain. Interest in the role of excitatory and inhibitory neurotransmission in depression has been stimulated by the discovery that ketamine, a glutamatergic NMDA receptor antagonist, exhibits rapid antidepressant activity through an apparently distinct mechanism from classic serotonergic antidepressant drugs (160). A recent meta-analysis of 49 ^1^H MRS glutamate studies found the strongest support for decreased glutamate levels in the medial PFC across 1,180 depressed vs. 1,066 healthy adults (161), with limited evidence supporting a similar pattern of glutamate dysregulation in depressed youth (162). Studies in animal models, meanwhile, suggest that GABA is involved in regulation of striatal dopamine release (163, 164), while both ^1^H MRS and lumbar puncture studies in adults have specifically reported GABA reductions in adults with melancholic depression (165, 166), a severe subtype of the disorder characterized by high anhedonia levels (167, 168). Using the dimensional approach advocated throughout this review, our laboratory sought to understand the possible link between GABA and reward dysfunction in youth. As spatial coverage using ^1^H MRS is limited, our investigations have focused on the ACC and the striatum, key reward-related regions frequently implicated in our studies of adolescent depression using other modalities.

Our first ^1^H MRS study utilized a case-control, cross-sectional design to examine GABA levels in a sample of 20 depressed and 21 healthy control adolescents (169). Analyses revealed that adolescents with depression had significantly lower ACC GABA than age-matched controls (*p* < 0.003) (169). When we split depressed adolescents into subgroups based on the presence of clinically significant anhedonia, only anhedonic subjects were found to have significantly lower GABA levels than controls. ACC GABA was also found to be negatively correlated with anhedonia severity in the depressed cohort (*r* = −0.50, *p* < 0.03) and the whole sample (*r* = −0.54, *p* < 0.001). These findings were the first evidence for an important association between ACC GABA and anhedonia severity in adolescent depression.

**Figure 10:**
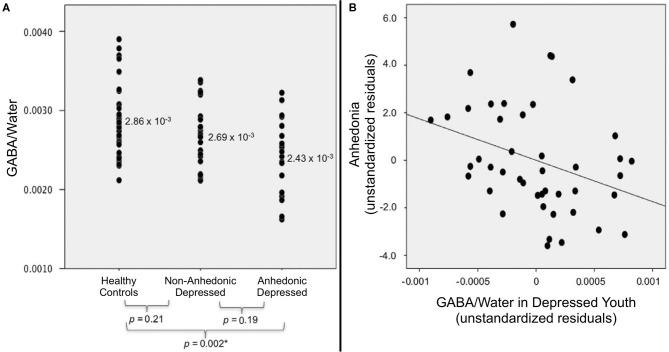
Relationship between anhedonia and ACC GABA levels in adolescents, measured by ^1^H MRS (standardized vs. water peak). **(A)** When depressed subjects were stratified into anhedonic (*n* = 19) and non-anhedonic (*n* = 25) subgroups, only anhedonic subjects differed significantly from healthy controls (*n* = 36). **(B)** Within the combined sample of depressed adolescents (*N* = 44), anhedonia was negatively associated with ACC GABA after controlling for depression severity (partial *r* = −0.33, *p* = 0.03). Excerpted from (170). ^*^Significant at the Šidák-corrected *p* < 0.05 level.

Our initial ACC GABA results were subsequently corroborated by a follow-up ^1^H MRS study in an expanded cohort, now including 44 depressed and 36 healthy control adolescents (170). Analyses were performed using analysis of covariance (ANCOVA) to control for age, sex, ethnicity, and cerebrospinal fluid content in the ACC voxel. Again, we found that ACC GABA levels were significantly lower in the depressed group (*p* = 0.003) relative to controls. When depressed subjects were classified based on anhedonia, analyses revealed a significant difference in ACC GABA levels across anhedonic depressed, non-anhedonic depressed, and healthy control adolescents (*p* = 0.003); as shown in Figure 10A, pairwise follow-up tests indicating that GABA levels were decreased only in the anhedonic depressed subgroup relative to controls (*p* = 0.002). Similar to our earlier results, anhedonia severity was inversely correlated with ACC GABA levels in the depressed group (Figure 10B), even when controlling for overall depression severity (*r* = −0.33, *p* = 0.03). These confirmatory findings support our conclusion that deficits in ACC GABA may serve as a biomarker for reward dysfunction in depressed adolescents.

Interestingly, we found the opposite relationship with striatal GABA levels in a related ^1^H MRS study of 20 depressed and 16 healthy control adolescents (171). In this case, our analyses revealed depressed youth to have higher GABA levels in the striatum compared to controls. Moreover, combining ^1^H MRS measures from both regions indicated that higher striatal GABA was associated with lower ACC GABA in adolescents with depression. While striatal GABA was not found to be associated with symptom severity, including anhedonia, the inverse relationship between ACC and striatal GABA levels hints at a possible protective role against the development of reward dysfunction. More than anything, this study highlights the need for further integrative ^1^H MRS research to fully map regional differences in GABA levels and their role in the etiology of adolescent depression.

**Figure 11:**
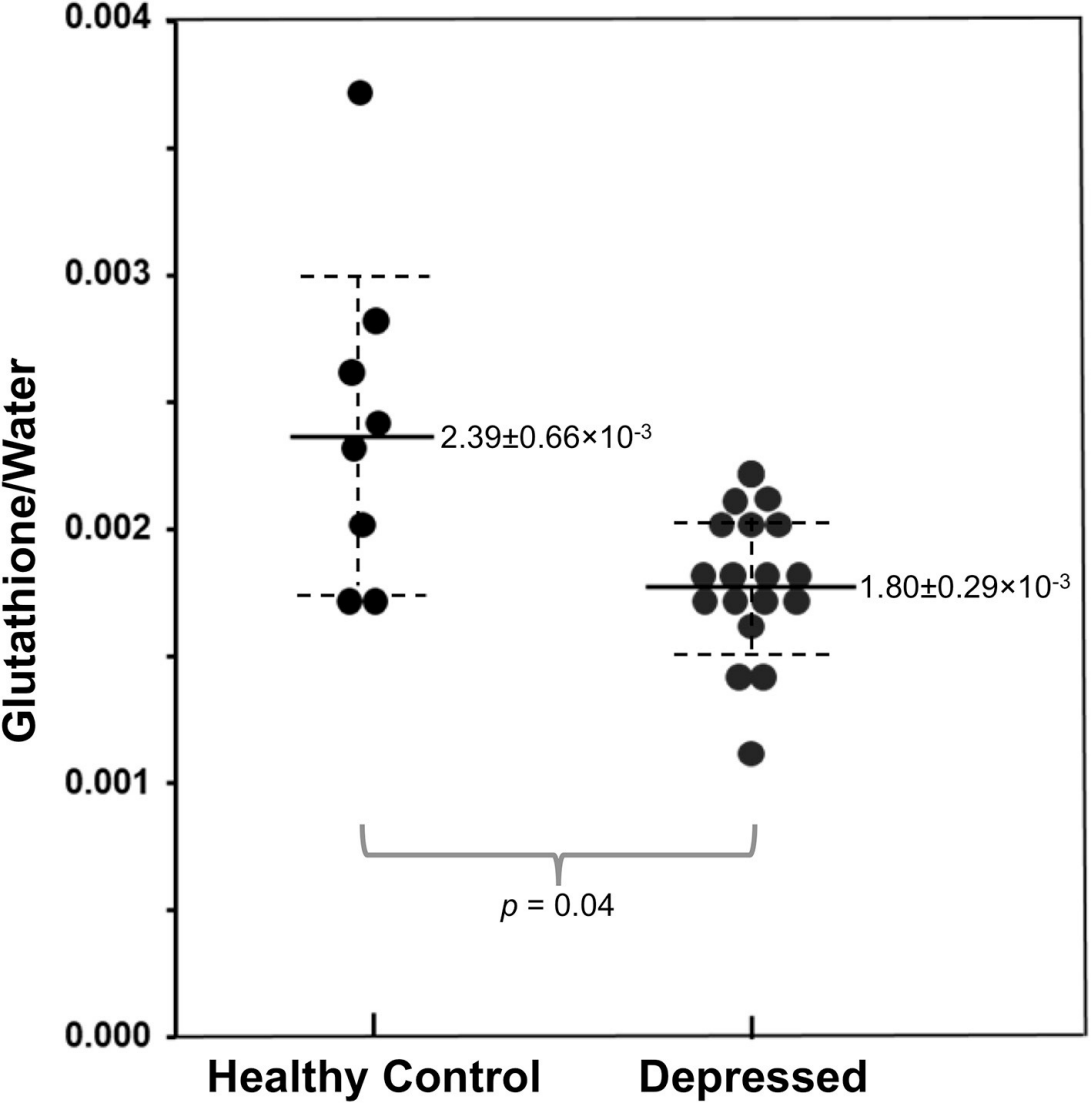
Reduced occipital glutathione relative to unsuppressed water signal in adolescents with major depressive disorder (*N* = 19) vs. healthy controls (*N* = 8), measured via ^1^H MRS. Adapted from (179). Solid and dashed lines represent mean and 95% confidence intervals, respectively.

In addition to studying GABA, our team has conducted preliminary ^1^H MRS investigations of glutathione, the primary antioxidant found in brain tissue. Glutathione plays a central role in cellular oxidative balance through non-enzymatic scavenging of free radicals and enzyme-catalyzed detoxification of hydrogen peroxide via glutathione peroxidase (172). Studies have documented decreased glutathione and glutathione peroxidase in both depressed humans and animal models of depression (173–177). In an exploratory study of 11 adults with major depressive disorder and 10 healthy controls, we found that anhedonia, but not fatigue, was negatively correlated with occipital glutathione levels indexed by ^1^H MRS (178). More recently, we published initial results from a study of occipital glutathione in 19 unmedicated adolescents (ages 12–21) with major depressive disorder and 8 age-matched healthy controls (179). Results indicated that cortical glutathione levels were significantly reduced in the depressed vs. control group (Figure 11), although correlations with anhedonia and overall depression severity did not meet statistical significance. Forthcoming, work from our group will evaluate glutathione and GABA levels in a larger cohort of adolescents to enable us to more definitively interpret the role of these important metabolites in adolescent depression and reward dysfunction.

### White Matter Integrity and Adolescent Depression Symptomatology

In 2013, our group published a pilot investigation into the role of white matter in adolescent depression using DWI (180). Specifically, diffusion-weighted data were collected at 3T using 1,000 s/mm^2^ gradients along 12 directions, with each direction sampled four times and averaged. Subjects comprised 17 depressed adolescents with a current major depressive episode of at least 8 weeks duration as well as 16 age-matched healthy controls; all participants were psychotropic-medication-naïve. Analyses were performed using FSL's Tract-Based Spatial Statistics pipeline (181) to model three principle diffusion directions per voxel and project the inferred white matter fibers onto a template “skeleton” representing major white matter tracts. Four standard measures derived from diffusion tensor modeling were examined: fractional anisotropy (FA), mean diffusivity (MD), radial diffusivity (RD), and axial diffusivity (AD). Results were reported at an exploratory threshold (uncorrected *p* < 0.001, *k* ≥ 10) using permutation-based non-parametric statistics.

Relative to healthy controls, we found that depressed adolescents had altered diffusivity metrics in four clusters: reduced anterior cingulum FA, anterior corona radiata AD, and posterior cingulum RD as well as increased posterior cingulum FA. In line with our other neuroimaging work and supporting our emphasis on dimensional symptom severity analyses, though, we found much more extensive associations with clinical symptomatology.

Depression severity correlated with a total of 16 clusters across diffusivity metrics, including negative correlations with FA in the anterior thalamic radiation near the left pallidum, genu of the corpus callosum near the ACC, and anterior cingulum bundle within the left precuneus; positive correlations with RD were observed in several overlapping clusters, including the anterior thalamic radiation bilaterally and the left genu of the corpus callosum.

Anhedonia severity was linked to altered diffusivity metric in 14 clusters, including reduced FA in the anterior limb of the internal capsule adjacent to the right basal ganglia; increased MD in the external capsule adjacent to the left putamen and in fibers projecting to the right OFC; and increased RD in the left anterior thalamic radiation, right anterior limb of the internal capsule, and projection fibers to the right OFC.

Unlike in our studies of clinical outcomes (section Anhedonia Predicts Worse Adolescent Depression Outcomes) and reward function using the RFT (section Task-Based Functional Magnetic Resonance Imaging), we also found associations between irritability scores and diffusivity metrics in a total of 14 clusters. These included positive correlations with MD in a cluster spanning the anterior corona radiata and anterior thalamic radiation near the left putamen, with RD in a cluster spanning the anterior limb of the internal capsule and anterior thalamic radiation near the right putamen, and with AD in the anterior corona radiata near the left putamen.

While preliminary, these results indicate that reduced white matter integrity in tracts projecting to or through major reward processing regions is associated with adolescent depression symptomatology. Unlike our resting-state and task-based fMRI results, we found that tracts with altered diffusivity metrics partially overlapped between different depression symptoms. This observation suggests that different disease mechanisms may be at work in gray vs. white matter, with the former exhibiting more specific abnormalities linked to reward dysfunction and the latter exhibiting more generalized loss of integrity in adolescent depression.

## Immune Function and Inflammation in Depression and Reward Dysfunction

Though the research discussed thus far emphasizes the neural underpinnings of depression and reward dysfunction, substantial evidence also implicates the immune system in these disease processes. Converging data from preclinical and clinical studies document that disturbances in reward circuitry are induced by inflammation (182–186), possibly reflecting an evolutionary adaptation to conserve energy and facilitate the healing process by limiting reward-seeking behavior (187–189). In animal models, immunological challenges such as exposure to lipopolysaccharide, a bacterial endotoxin and potent inflammatory agent, result in “sickness behavior” characterized by decreased social exploration, sleep disorders, and, in female rats, inhibition of sexual behavior (190–195). In other words, the sickness behavior phenotype reflects broad-based deficits in reward processing (187) and mirrors many aspects of depression phenomenology in humans (see section Shortcomings of the Categorical Diagnostic System).

At the same time, mounting evidence has documented activation of oxidative and nitrosative pathways, which are known to be induced by inflammatory responses, in depression. Reported abnormalities include decreased antioxidant levels, lowered antioxidant enzyme activity, increased circulatory oxygen radicals, and oxidative damage to lipids in depressed adults and in animal models of depression (196). As noted in section Neurochemical Abnormalities Revealed by Magnetic Resonance Spectroscopy, our group found a negative association between cortical glutathione and anhedonia in a pilot ^1^H MRS study in adults (178). Simultaneously, expression of multiple neurotrophic factors (e.g., brain derived neurotrophic factor, BCL-2 proteins) is suppressed in depression (197).

Based on these observations, a leading theory posits that the pathophysiology of depression and reward dysfunction can be traced an imbalance between neurotrophic and neurotoxic factors, resulting in a cumulative loss or atrophy of neurons and glia (197–201). Supporting evidence includes significant volume reductions in brain volume, altered metabolic rates, and neurochemical abnormalities in specific brain regions including the PFC, amygdala, hippocampus, and basal ganglia, along with disturbances in limbic-cortical-striatal-pallidal-thalamic circuits (202). Postmortem studies provide additional evidence of neurodegenerative processes in depression, with decreased cortical thickness, neuronal size, and neuronal/glial density reported in the left rostral OFC, ACC, dorsolateral PFC, and hippocampus (203). However, the direct mechanisms giving rise to the proposed neurotrophic/neurotoxic imbalance in depression remain largely unknown, especially during the critical neurodevelopmental period of adolescence (section Reward Dysfunction as a Promising Predictor of Adolescent Depression Trajectory). Here, we describe our work to elucidate key metabolic and immunological pathways and their role in reward dysfunction early in the course of illness.

### The Kynurenine Pathway Links Immune and Reward Function

**Figure 12:**
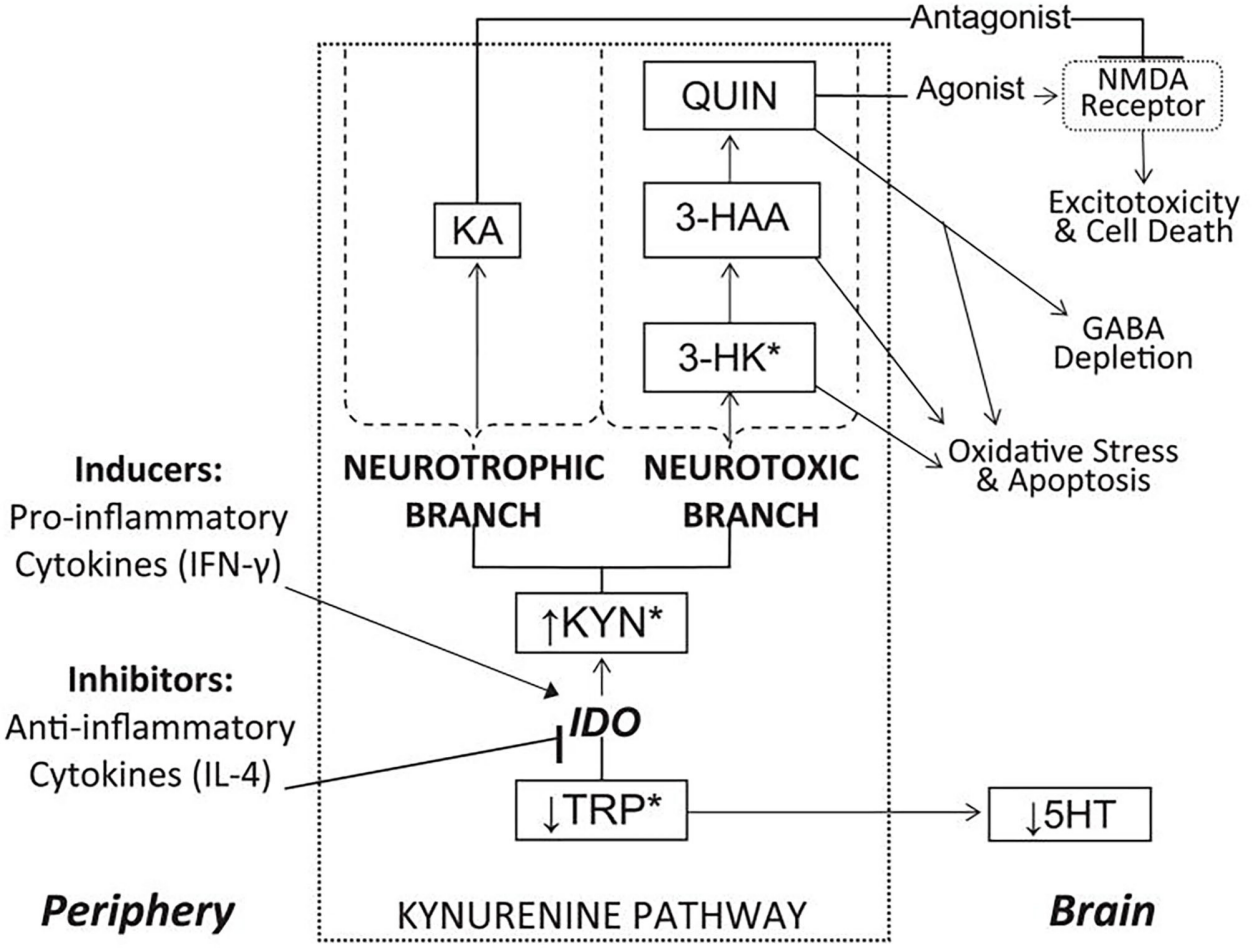
Overview of the kynurenine pathway and its position as a link between peripheral inflammation and central reward circuitry disruption. See list of acronyms at the start of main text. Reprinted from (204). ^*^Metabolites capable of crossing the blood-brain barrier.

Our laboratory has conducted extensive research into the kynurenine pathway (204–208), a series of catabolic reactions that accounts for the breakdown of approximately 99% of free tryptophan (TRP), the rate-limiting amino acid precursor in serotonin (5-HT) synthesis (209). Due to the excitotoxic properties of several biomolecules synthesized via the kynurenine pathway, together with the reduction in TRP availability for 5-HT synthesis, the kynurenine pathway has been hypothesized to play an important role in linking peripheral inflammatory processes to central nervous system oxidative stress, atrophy, and cell death in depression and other disorders (210–212). The major enzymes, metabolites, and branches of the kynurenine pathway, as well as proposed links to neuroinflammatory disease processes, are illustrated in Figure 12.

The rate-limiting enzyme in the pathway is indoleamine 2,3-dioxygenase (IDO), which is induced by pro-inflammatory cytokines (213) and metabolizes TRP into kynurenine (KYN). KYN is then compartmentally metabolized in the brain via two major branches. In the neurotoxic branch, KYN is sequentially converted into 3-hydroxykynurenine (3-HK) and 3-hydroxyanthranilic acid (3-HAA), neurotoxins that contribute to free radical generation, *en route* to forming quinolinic acid (QUIN), an excitotoxic agonist of glutamatergic N-methyl-D-aspartate (NMDA) receptors (214). Alternatively, in the neurotrophic branch, KYN is metabolized into kynurenic acid (KA), an NMDA receptor antagonist with neuroprotective properties (215). Thus, induction of kynurenine pathway metabolism, especially the neurotoxic branch, may contribute to multiple neurodegenerative mechanisms in depression.

Converging lines of research have indeed linked increased IDO activity to anhedonia and reward dysfunction (216, 217). In mice, peripheral inhibition of IDO has been shown to block transcription of IDO in the brain and prevent the development of depression- and anhedonia-like behaviors following immunological stimulation (218, 219). In humans, clinical studies have reported increased urinary excretion of KYN and 3-HK in anhedonic depressed women (220) as well as relationships between kynurenine pathway activity and anhedonia in adults with depression (221–224). Moreover, we recently reported increased kynurenine pathway activity in suicidal depressed youth, further emphasizing the clinical significance of TRP metabolism (207).

#### The Kynurenine Pathway in Adolescent Depression and Anhedonia

An earlier series of studies by our group provided the first evidence of kynurenine pathway activation in adolescent depression and specifically in anhedonia. Our first study examined a cohort of 50 adolescents with major depressive disorder and 22 healthy controls, with the depressed cohort divided into 20 melancholic subjects, characterized by more severe illness and prominent anhedonia, and 30 non-melancholic subjects (204). We found that adolescents with depression had significantly lower plasma TRP concentrations and higher KYN/TRP ratios, a proxy for IDO activity, than either non-melancholic depressed or healthy control subjects. Moreover, within the melancholic cohort, KYN levels were negatively correlated with depression severity, while 3-HAA/KYN ratios were positively associated with depression scores, indicating preferential engagement of the neurotoxic KYN → 3-HK → 3-HAA branch of the kynurenine pathway.

Building on these results, we subsequently examined the possible role of the kynurenine pathway in anhedonia, the hallmark symptom of melancholia (206). In a sample of 36 adolescents with major depressive disorder, including 22 medication-free participants and 20 healthy controls, we found that plasma concentrations of kynurenine pathway metabolites correlated with anhedonia severity while controlling for overall depression severity. Interestingly, IDO activity (i.e., KYN/TRP ratio) positively correlated with anhedonia severity in medication-free adolescents with depression alone (*r* = 0.42, *p* = 0.05) and in combination with controls (*r* = 0.44, *p* = 0.004), but this correlation was reduced when the combined sample included medicated depressed subjects (*r* = 0.30, *p* = 0.02) and did not reach significance in the depressed cohort alone when medicated subjects were included (*r* = 0.31, *p* = 0.06). This study was the first to link reward dysfunction to kynurenine pathway induction in youth. Additionally, our findings highlight the important, but frequently underappreciated, impact that treatment can have on biological measures in psychiatric research, suggesting that psychotropic medication may have a normalizing effect on the neurometabolic abnormalities observed in unmedicated participants.

#### Preliminary Findings From Kynurenine Pathway Neuroimaging

Several of our neuroimaging studies further corroborate the link between immunological disruptions and reward deficits in adolescents. In a pilot study of seven melancholic depressed, seven non-melancholic depressed, and six healthy control adolescents (205), we found that peripheral KYN levels correlated with total choline, a spectroscopic biomarker for cell membrane turnover, in the right caudate (*rho* = 0.93, *p*_*Bonferroni*_ = 0.03), while peripheral 3-HAA correlated with total choline in the left putamen (*rho* = 0.96, *p*_*Bonferroni*_ = 0.006) only within the melancholic (i.e., highly anhedonic) depressed subgroup. While the small sample of this preliminary investigation is obviously a limitation, the magnitude of the observed correlations was remarkably high, suggesting a possible link between the global TRP metabolism and regional cell membrane metabolism within the striatum. A more recent pilot analysis of resting-state fMRI and kynurenine pathway metabolites in 14 unmedicated adolescents with depression and seven healthy controls similarly found preliminary evidence of associations between several kynurenine pathway metabolites and RSFC within reward- and salience-related neurocircuitry (208).

### Comprehensive Cytokine Profiling Reveals Extensive Associations With Anhedonia

As the primary signaling molecules mediating human immune response, cytokines have been frequent target of investigation across disease states, and the link between elevated levels of pro-inflammatory cytokines and depression is robustly documented (201, 225, 226). This includes a substantial body of evidence supporting a direct, causal link between exposure to pro-inflammatory cytokines or inflammatory triggers and reward dysfunction. In rhesus monkeys, chronic administration of interferon-alpha (IFN-α), a major pro-inflammatory cytokine, results in decreased dopaminergic neurotransmission along with anhedonia-like behavior (227). Extremely similar results are described in humans treated with IFN-α as part of immunotherapy regimes (228), who report a “flu-like”' syndrome characterized by anhedonia, fatigue, anorexia, and hypersomnia—again, all hallmarks of depression. Another study of healthy individuals who received lipopolysaccharide vs. saline placebo injections likewise reported that exposure to the endotoxin induced motivational changes in a behavioral task (229). However, the vast majority of clinical evidence supporting a link between inflammation and depression has come from studies in adult cohorts, and the relationship between these phenomena in pediatric populations remains less clear (226).

To address this gap, initial studies by our group examined plasma levels of major pro-inflammatory cytokines in the context of adolescent depression. We documented significantly higher levels of interferon-gamma (IFN-γ) in medically healthy adolescents with major depressive disorder compared to healthy controls (230, 231); unexpectedly, however, we found that suicidal depressed adolescents had decreased levels of tumor necrosis factor-alpha (TNF-α) compared to non-suicidal adolescents with depression (231). Building on this initial work, we performed a novel *in vitro* experiment using peripheral blood mononuclear cells to study the role of cytokine induction in the emergence of adolescent depression symptomatology without exposing this vulnerable population to a potentially aggravating immunological challenge (232). These cells share a common mesodermal lineage with microglia and have an overlapping gene expression profile, thus expressing many of the same surface receptor and signaling proteins as their inaccessible counterparts in the brain (233, 234). Blood samples were obtained from a clinical sample of 54 adolescents with predominantly mood and anxiety symptoms as well as 22 healthy control adolescents. Peripheral blood mononuclear cells were cultured for 6 h in the presence of lipopolysaccharide, after which the supernatant fluid was collected and assayed. To better capture the complex, multivariate nature of the immune system, comprehensive panels were performed using multiplex fluorescence assays to quantify 41 distinct pro- and anti-inflammatory cytokines. Analyses were controlled for multiple comparisons across all 41 measures (*p*_*FDR*_ < 0.05) as well as overall depression severity, body mass index, age, and sex. Even at this stringent threshold, we found that anhedonia was positively correlated (*rho* = 0.33–0.57) with 19 of the cytokines included in our panel. Cytokines linked to anhedonia were primarily pro-inflammatory and/or hematopoietic growth factors (*N* = 15), with a small number of chemokines (*N* = 2) and anti-inflammatory cytokines (*N* = 2) also identified. In line with our other dimensional work, no significant group differences were observed between depressed adolescents and healthy controls, and no significant association was detected with any other clinical measure (i.e., depression severity, anxiety, suicidality, fatigue). These findings provide strong evidence that inflammatory responses are tied to reward deficits in adolescents regardless of diagnostic status and again showcase the central role of reward dysfunction in early depression symptomatology.

### Data-Driven Analysis of Inflammatory Factors and Reward Function

Neuroimaging work further documents the specific effects of inflammatory processes within the dopaminergic reward circuitry. Several positron emission tomography (PET) studies in adults have demonstrated changes in striatal glucose metabolism subsequent to immunotherapy (235, 236). Using fMRI, decreased striatal activation in response to rewarding stimuli was reported in patients undergoing treatment with IFN-α (237). Similarly, exposure to bacterial endotoxins in healthy volunteers resulted in decreased ventral striatum activation during a reward task (238) but increased pain sensitivity, elevated activation in the anterior insula (a region involved in pain perception), and reduced activation in the rostral ACC (a region involved in pain regulation) during a noxious pressure paradigm (239). Typhoid vaccination in healthy volunteers has also been shown to alter activity in the substantia nigra, part of the dopaminergic midbrain, in relation to psychomotor slowing (240). Additionally, multiple neuroanatomical studies in depressed adults have documented relationships between peripheral kynurenine pathway metabolites and volume changes in reward-related brain regions (241–244).

**Figure 13:**
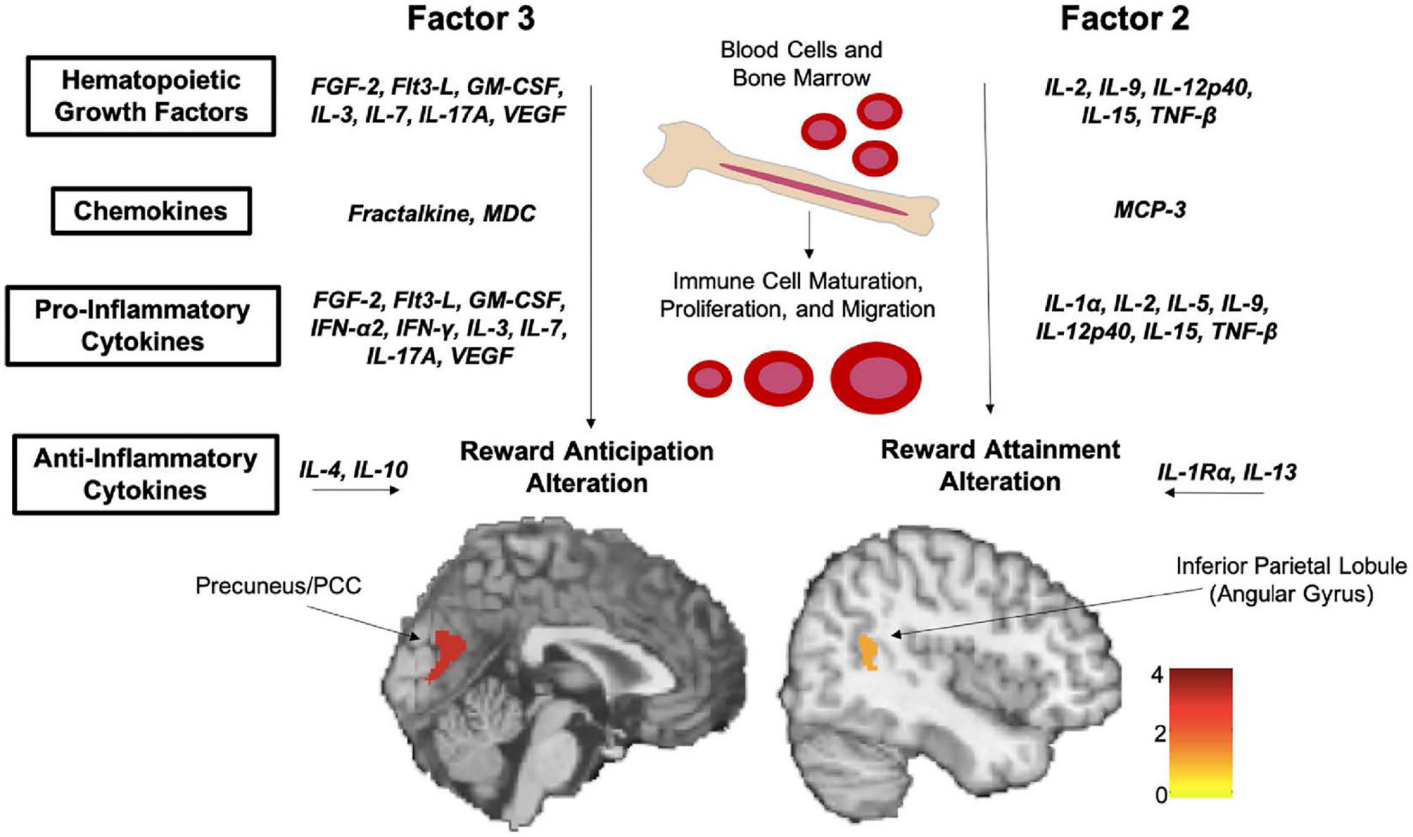
Two factors identified by data-driven principal component analysis (PCA) of cytokine panels were found to significantly correlate with neural reward function in adolescents. Both factors were dominated by markers of inflammation and hematopoietic growth. Reprinted from (245). Scale bar indicates *z*-score.

One of our latest projects combined elements of our detailed cytokine panel analysis, described in section Comprehensive Cytokine Profiling Reveals Extensive Associations With Anhedonia, with our task-based fMRI studies using the RFT, presented in section Task-Based Functional Magnetic Resonance Imaging, to map between inflammation and neural reward processes in adolescents (245). Subjects consisted of 34 unmedicated adolescents with significant clinical symptoms predominantly related to mood and anxiety conditions and 12 age-matched healthy controls who completed both a blood draw and the RFT fMRI task. Multiplex fluorescent panels to measure 41 cytokines and fMRI analyses to model activation during reward anticipation and reward attainment were performed as described above. However, to address the high degree of correlation between many cytokines and limit the issue of multiple comparisons, we performed an additional dimensionality reduction step using factor analysis, a data-driven technique to identify shared sources of variance in complex, multivariate datasets. This approach yielded four inflammatory factors that together explained 76.4% of variance across all cytokines. As shown in Figure 13, one of these factors was found to negatively correlate with activation in three bilateral posterior cingulate/inferior precuneus clusters during reward anticipation, while another was negatively correlated with activation in a single right angular gyrus cluster during reward attainment (all whole-brain *p*_*FWE*_ < 0.05). Remarkably, 16 of the 19 cytokines found to be associated with anhedonia severity in our earlier dimensional study (232) were also identified in this fMRI analysis, implying that a common set of inflammatory modulators influence both clinical and neural reward function.

## Conclusions and Future Directions

The ongoing transition from traditional, diagnosis-based research to modern, dimensional paradigms marks a sea change in psychiatry and holds great promise to overcome longstanding barriers to understanding depression. Our group has pursued this dimensional approach to examine anhedonia as a complex behavioral construct that reflects alterations in developing reward circuitry. Early on, we showed that anhedonia severity varied widely and was associated with worse outcomes in adolescents with moderate-to-severe depression. Our subsequent clinical investigations have confirmed and extended this conclusion, indicating that higher anhedonia specifically predicted future depression severity and suicidality. At the same time, we documented substantial individual variation in anhedonia severity that cut across disorders and was present even in healthy adolescents. Our research program has therefore targeted anhedonia as a window to studying the neurobiological underpinnings of reward dysfunction in youth.

To this end, we employ an array of *in vivo* neuroimaging modalities to examine the mechanisms driving reward dysfunction in adolescent depression and across psychiatric conditions. With novel fMRI tasks, we were able to parse distinct phases of reward processing and related cognitive functions in both clinical and healthy adolescent cohorts. We found that reward anticipation, reward attainment, and reward prediction errors differentially elicited brain activity within and beyond the dopaminergic reward system. Subsequent research revealed that altered activation during these reward processes was linked to future reward deficits in the form of anhedonia. Using complementary resting-state fMRI techniques to map subcortical RSFC networks, we identified circuit-level alterations that were associated with anhedonia and depression severity as well as with subclinical depression. Bridging task-based and resting-state fMRI with data-driven graph theoretical analyses, we detailed differential relationships between anticipatory vs. consummatory anhedonia with RSFC features of whole-brain and reward-task-derived networks, highlighting the need for detailed dimensional measures to fully elucidate reward-related constructs. In conjunction with fMRI, we employed ^1^H MRS to measure regional GABA levels within key reward regions, providing biochemical evidence linking cortical GABA deficits to clinical reward deficits and more severe depression in youth. Most importantly, findings across different neuroimaging modalities converged on a relatively small number of structures, namely the ACC and striatum, that appear to play a disproportionate role in the emergence of depression symptomatology in youth.

Our group has also systematically examined inflammation and its contributions to pediatric reward dysfunction. We found evidence connecting activation of the kynurenine pathway, which degrades tryptophan into a series of neurometabolically active toxic species, to reward deficits (anhedonia), resting-state network anomalies, and altered neurochemical profiles. Employing detailed cytokine panels to capture a wide spectrum of immune biomarkers as well as an *in vitro* immunological challenge to induce inflammatory responses, we documented a unique relationship between anhedonia and immune activity in adolescents. Capitalizing on our concurrent collection of neuroimaging data in the same cohort, we employed a data-driven dimensionality reduction and identified a pair of immune factors that correlated with neural measures of reward function. In summary, the use of dimensional investigative techniques together with rigorous methodology and an integrative data collection strategy has enabled our group to characterize key aspects of reward dysfunction in youth that cut across disorders.

It is important to note that, while the dimensional approach offers many advantages over categorical diagnostics for understanding disease mechanisms, it also presents its own challenges and limitations. Of particular concern given our focus on reward dysfunction are the nuances of “anhedonia.” While far more specific than “depression,” insofar as anhedonia represents a well-defined clinical phenomenon (i.e., reduced capacity to experience pleasure), efforts to develop reliable anhedonia scales indicate that it is not a monolithic construct. Indeed, the TEPS questionnaire employed in many of our studies is subdivided into items related to reward anticipation (i.e., pleasure at the thought of future rewards) vs. reward consumption (i.e., pleasure experienced when a reward is obtained). Subsequent work has suggested that two subscales may be inadequate to fully capture anhedonia phenomenology, particularly in cross-cultural settings. The TEPS was originally developed by researchers in the University of California system and validated based on responses of undergraduate volunteers from the Berkeley campus (48). A team of researchers from the University of the Chinese Academy of Sciences, however, found that a four-factor model that further subdivided pleasure experience into abstract components (i.e., items regarding beliefs about pleasure in general) and contextual components (i.e., items regarding specific pleasurable events) was a better fit for Chinese undergraduate students as well as ethnically Asian undergraduate students from an American sample, whereas the both models fit the overall American model equally well (51). Conversely, another group from the University of Melbourne examined TEPS responses from university students in the United Kingdom and Australia and reported that, although a two-factor model did not adequately model the response data, this was due to the high correlation between items across subscales, and a two-factor solution still outperformed the four-factor version (53). At present, the question of how to best discriminate between aspects of reward dysfunction remains unresolved; it is likely that additional, objective metrics such as the Probabilistic Reward Task developed by Diego Pizzagalli and colleagues (246) are needed to supplement subjective questionnaires and reliably quantify hedonic capacity. A related concern is that reward dysfunction may have distinct etiological mechanisms in different psychiatric contexts. It is possible, e.g., that anhedonia associated with chronic substance abuse may result from unique circuit-level changes compared to anhedonia associated with depression, which presumably arises from issues with endogenous reward signaling. Finally, while this review has attempted to illustrate the emerging discipline of dimensional psychiatric research by highlighting key findings from across the field in conjunction with our own work, it should not be taken as a comprehensive overview of all the important and exciting work being done to map the origins of reward dysfunction in adolescents.

Moving forward, we aim to build on the foundation of knowledge detailed above and extend our work further by incorporating powerful new resources that are just becoming available to the community through large, multi-site neuroimaging initiatives. Prominent examples include the Nathan Kline Institute-Rockland Sample (NKI-RS) and HCP Lifespan Initiative, which aims to create large (*N* > 1,000) community profiles across age groups (247–249), as well as the Adolescent Brain Cognitive Development (ABCD) study, which aims to track >10,000 participants from pre-adolescence (age ~10) to early adulthood (age ~18) in the largest multi-site study currently operating in the United States (250). These projects are designed to overcome the inherent limitations of smaller-scale cross-sectional and longitudinal studies in childhood or adolescence, which frequently suffer from insufficient power, high attrition, and inadequate follow-up periods, as well as study designs being restricted to a specific neural system (251). The dynamic and sometimes ephemeral nature of depression symptomatology in adolescents makes it especially critical to compile multiple observations within a large and diverse cohort over time. Incorporating high-quality data from these public sources in conjunction with our ongoing, targeted investigations provides an exciting new avenue to pursue the neurobiological origins of reward dysfunction in youth and ultimately identify reliable predictors of illness trajectory into adulthood.

## Author Contributions

AW, BE, RT, TN, and VG: conceptualization, writing—original draft, and writing—review and editing. BE: visualization. VG, BE, and TN: funding acquisition. All authors contributed to the article and approved the submitted version.

## Conflict of Interest

The authors declare that the research was conducted in the absence of any commercial or financial relationships that could be construed as a potential conflict of interest.

## Publisher's Note

All claims expressed in this article are solely those of the authors and do not necessarily represent those of their affiliated organizations, or those of the publisher, the editors and the reviewers. Any product that may be evaluated in this article, or claim that may be made by its manufacturer, is not guaranteed or endorsed by the publisher.

## Abbreviations

3-HAA: 3-hydroxyanthranilic acid
3-HK: 3-hydroxykynurenine
5-HT: serotonin
ABCD: Adolescent Brain Cognitive Development
ACC: anterior cingulate cortex
AD: axial diffusivity
ANCOVA: analysis of covariance
BOLD: blood-oxygenation-level-dependent
CDRS-R: Children's Depression Rating Scale-Revised
dACC: dorsal anterior cingulate cortex
dlPFC: dorsolateral prefrontal cortex
DSM: Diagnostic and Statistical Manual of Mental Disorders
DWI: diffusion-weighted imaging
EEG: electroencephalography
FA: fractional anisotropy
fMRI: functional magnetic resonance imaging
FDR: false discovery rate
FWE: family-wise error
GABA: γ-aminobutyric acid
^1^H MRS: proton magnetic resonance spectroscopy
HCP: Human Connectome Project
ICD: International Statistical Classification of Diseases and Related Health Problems
IDO: indoleamine 2,3-dioxygenase
IFN-α: interferon-alpha
IFN-γ: interferon-gamma
KA: kynurenic acid
KYN: kynurenine
MD: mean diffusivity
MSM: multimodal surface matching
NIH: National Institutes of Health
NIMH: National Institute of Mental Health
NKI-RS: Nathan Kline Institute-Rockland Sample
NMDA: N-methyl-D-aspartate
OFC: orbitofrontal cortex
PET: positron emission tomography
PFC: prefrontal cortex
QUIN: quinolinic acid
RD: radial diffusivity
RDoC: Research Domain Criteria
RFT: reward flanker task
RSFC: resting state functional connectivity
sgACC: subgenual anterior cingulate cortex
SHAPS: Snaith-Hamilton Pleasure Scale
T1w: T1-weighted
T2w: T2-weighted
TADS: Treatment for Adolescents with Depression Study
TEPS: Temporal Experience of Pleasure Scale
TFCE: threshold-free cluster enhancement
TNF-α: tumor necrosis factor-alpha
TORDIA: Treatment of Resistant Depression in Adolescents
TRP: tryptophan.
